# Efficient Concentration and Complete Destruction of Short‐Chain and Emerging PFAS in Contaminated Water via Integrated Interface Engineering‐Enhanced Carbon Felt Sorption and Photochemical Processes

**DOI:** 10.1002/advs.76133

**Published:** 2026-06-15

**Authors:** Hao Yu, Jialei Guo, Peng Zhang, Hongyi Li, Feng He, Hongwen Sun

**Affiliations:** ^1^ MOE Key Laboratory of Pollution Processes and Environmental Criteria, Tianjin Key Laboratory of Environmental Technology For Complex Trans‐Media Pollution, College of Environmental Science and Engineering Nankai University Tianjin China; ^2^ School of Petrochemical Engineering & Environment Zhejiang Ocean University Zhoushan China; ^3^ Haihe Laboratory of Sustainable Chemical Transformations Tianjin China; ^4^ Institute of Environmental Processes and Pollution Control and School of Environment and Ecology Jiangnan University Wuxi China

**Keywords:** adsorption, carbon felt, interface engineering strengthening, photochemical destruction, short‐chain pfas

## Abstract

Short‐chain and emerging per‐ and polyfluoroalkyl substances (PFAS) pose a significant threat to water security due to their high mobility and environmental persistence. Current strategies relying solely on adsorption or degradation remain inefficient and incomplete solutions. Herein, a closed‐loop “Concentrate‐&‐Destroy” strategy integrating an interface engineering‐enhanced sorbent of the polypyrrole (PPy) nanostructured coating carbon felt (PPy@P‐CF) with Cu^2+^‐mediated photochemical destruction is presented. This designed PPy@P‐CF enabled rapid and efficient adsorption of short‐chain PFAS (C2‐C4) and emerging GenX, with equilibrium achieved within 300 s and maximum sorption capacities reaching 283.2–572.1 mg g^−1^, surpassing those of granular activated carbon (GAC) and CF by over 21 and 5 times, respectively, under identical conditions. Dynamic column tests demonstrated a 5‐fold improvement in breakthrough volume (exceeding 4000 bed volumes) compared to GAC and CF columns. Furthermore, the spent PPy@P‐CF can be regenerated by solvent washing for reusability, and Cu^2+^‐mediated photochemical degradation achieved complete defluorination of concentrated PFAS in eluate within 5 h by a ligand‐to‐metal charge transfer process. In practical applications, a bulk PPy@P‐CF module achieved > 96% removal of 27 diverse PFAS from real contaminated water, followed by complete photochemical defluorination. This integrated approach provides an adoptable solution for eliminating persistent short‐chain and emerging PFAS.

## Graphical abstract

A closed‐loop “Concentrate‐Destroy” strategy is presented for completely eliminating short‐chain and emerging PFAS, in which the new 3D PPy@P‐CF is used for efficiently concentrating PFAS and Cu^2+^‐mediated photodegradation is applied for the completely destroying PFAS in the sorbent regeneration eluent. This integrated approach provides an adoptable solution for eliminating persistent short‐chain and emerging PFAS in the contaminated water.

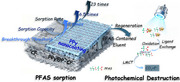

## 1 Introduction

Per‐ and polyfluoroalkyl substances (PFAS), comprising over 16 000 synthetic compounds, have been extensively utilized in industrial and consumer applications since the mid‐20th century due to their unique chemical and thermal stability [1, 2, 3]. The strong C‐F bonds (536 kJ mol^−1^) render PFAS highly persistent, bioaccumulative, and ubiquitous in global natural and wastewater systems, with concentrations detected from ng/L to µg/L levels [4, 5, 6, 7]. Mounting epidemiological evidence links PFAS exposure to severe health risks, including neurotoxicity, immunotoxicity, and carcinogenicity [8, 9, 10]. In response, regulatory limits have become increasingly stringent; for instance, on April 10, 2024, the United States Environmental Protection Agency (U.S. EPA) established maximum contaminant levels of 4.0 ng L^−1^ for perfluorooctanoic acid (PFOA) and 10.0 ng L^−1^ for hexafluoro‐propylene oxide‐dimer acid (HFPO‐DA, trade name GenX, an emerging polyfluorinated alternative) in drinking water [11]. Compounding this challenge is the rising prevalence of short‐chain (C < 6) and emerging PFAS, which exhibit higher mobility and resistance to conventional removal technologies due to their hydrophilic nature [12, 13, 14].

Various strategies have been developed to address PFAS contamination in water, such as sorption, photocatalysis, electrochemical oxidation, advanced oxidation and reduction, and thermochemical treatments [15, 16, 17]. Among these, sorption is the most widely implemented for its operational simplicity and potential to break the “forever chemical” cycle [18, 19]. However, conventional sorbents, including granular activated carbon (GAC) and ion‐exchange resins (IERs), are predominantly effective for long‐chain PFAS (C ≥8) but exhibit limited affinity for short‐chain and emerging congeners. This limitation is particularly pronounced for ultrashort‐chain trifluoroacetic acid (TFA, C2) in complex water matrices, due to its enhanced hydrophilicity, high mobility, and small molecular size [20, 21, 22]. For example, at environmentally relevant concentrations ([GAC] = 10 mg L^−1^, [PFAS]_0_ = 1 µg L^−1^), GAC achieves a removal efficiency of less than 40% for TFA and perfluorobutanoic acid (PFBA, C4), compared to over 60% for PFOA (C8) [23]. Thus, the development of high‐capacity, rapid sorbents capable of capturing diverse short‐chain and emerging PFAS from real water systems remains a critical gap. Moreover, while sorption serves to concentrate PFAS, it does not achieve their destruction, generating secondary waste streams, such as highly contaminated regenerant eluents, that pose significant disposal challenges [24, 25, 26, 27]. Recently, in response to the limitations of physical sequestration, photocatalytic degradation has emerged as a promising destructive technology, leveraging light‐induced reactive species (e.g., hydroxyl radicals (HO•), holes (h^+^)), hydrated electrons (e_aq_^−^)) to mineralize PFAS under ambient conditions [28, 29, 30]. Notably, Cu^2+^‐mediated photochemical degradation exploits the coordination between PFAS carboxylates and the metal center, enabling ligand‐to‐metal charge transfer (LMCT) under 365 nm LED irradiation. This approach provides a milder and more energy‐efficient alternative to conventional catalytic methods, characterized by low energy demand and simple operation [28, 29, 30]. Herein, we propose an integrated treatment strategy that couples sorption‐based concentration with photocatalysis destroying. Sorption preconcentrates trace levels of PFAS from large‐volume water into a small‐volume eluent, thereby enabling highly efficient and energy‐efficient photodegradation [31, 32]. Crucially, by irradiating only the concentrated eluent, rather than the entire bulk water volume, the process achieves substantial energy savings without compromising degradation performance [31, 32].

Three‐dimensional (3D) flexible carbon materials such as carbon felt (CF) offer structural advantages, including interconnected macroporous networks, mechanical robustness, and customizable geometry, making them increasingly attractive for applications in water treatment across diverse scenarios [33, 34]. However, their inherent hydrophobicity and negatively charged surfaces limit effective interactions with hydrophilic and electronegative short‐chain PFAS, such as TFA and PFBA. Our prior work demonstrated that modifying biochar surfaces with polypyrrole (PPy) nanoparticles can enhance PFAS sorption via electrostatic interactions [35]. Instead, the enhancement of PFAS removal sacrificed most pores of the biochar (specific surface area (SSA) decreasing 300% after modification), which limited the potential sorption capacity of PPy‐modified biochar. Therefore, we hypothesize that interfacial engineering that coating the 3D carbon material with a polyaniline (PPy) nanofilm can simultaneously preserve its intrinsic porous architecture and precisely modulate surface chemistry to enhance localized electric fields. This dual functionality enables efficient preconcentration of diverse PFAS from real‐world contaminated water, thereby facilitating subsequent photocatalytic degradation of PFAS‐concentrated eluents regenerated from spent sorbents and advancing the scalability of the integrated process for large‐scale water treatment.

**FIGURE 1:**
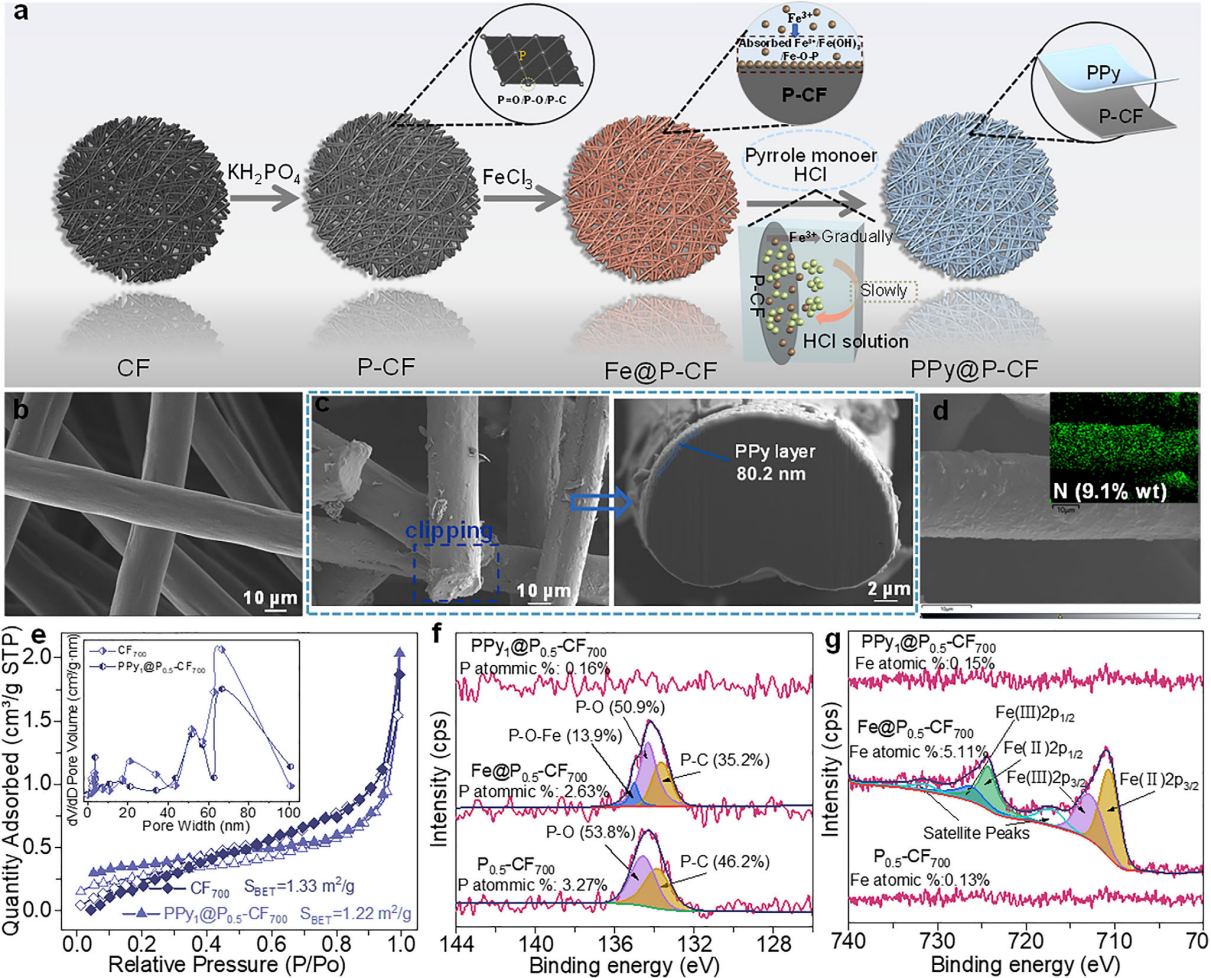
Schematic diagram of the preparation of the PPy@P‐CF (a); SEM images of pristine CF_700_ (b); FIB‐SEM images of cross‐sections of PPy_1_@P_0.5_‐CF_700_ (c); SEM images and EDS mapping of PPy_1_@P_0.5_‐CF_700_ (d); Nitrogen adsorption‐desorption isotherms and pore size distributions of CF_700_ and PPy_1_@P_0.5_‐CF_700_ (e); Fe2p XPS spectra (f); P2p XPS spectra (g).

In the current study, we address the critical challenge of eliminating ultrashort‐chain, short‐chain, and emerging PFAS from contaminated water by introducing an integrated interface engineering‐enhanced CF sorbent coupled with photochemical degradation. We developed a PPy nanostructured coating on CF (PPy@P‐CF; Figure 1a) to overcome the limitations of conventional adsorbents, which exhibit poor affinity for hydrophilic and structurally complex PFAS. The goal of this study was to efficiently concentrate and completely destroy short‐chain and emerging PFAS from contaminated water. A systematic evaluation was conducted, including comprehensive material characterization, batch and dynamic column tests, and assessment of 27 additional PFAS, covering both legacy and emerging PFAS identified via nontarget analysis from real PFAS‐contaminated natural water and wastewater. Beyond superior adsorption performance, this work demonstrates a closed‐loop “Concentrate‐&‐Destroy” strategy: post‐sorption, PFAS‐laden PPy@P‐CF was efficiently regenerated, and the concentrated PFAS in eluate was completely degraded and defluorinated via a Cu^2+^‐mediated photochemical process under mild conditions. Mechanistic insights derived from molecular dynamics simulation (MDS) and spectroscopic analyses elucidate the roles of pore confinement, electrostatic attraction, and interfacial mass transfer in the adsorption process. By synergizing efficient PFAS concentration with complete mineralization, this study offers an adoptable technological pathway to mitigate the global burden of persistent and mobile PFAS in water bodies.

## 2 Results and Discussion

### 2.1 Surface Electric Field Strengthening via Interface Engineering and Pore Preservation

An interface engineering‐enhanced strategy via PPy nanocoating was proposed to strengthen the surface electric field while preserving the porous structure of CF, as illustrated by the case of PPy@P‐CF in Figure 1a. Specifically, CF_700_ consists of interwoven carbon fibers with a smooth and flat surface, forming a nest‐like architecture with abundant macropores (Figure 1b). In contrast, the surface of the carbon fibers in PPy‐coated CF_700_ (PPy_1_@P_0.5_‐CF_700_) became noticeably rougher, though the overall morphology remained consistent with that of CF_700_ (Figure 1c). Furthermore, a distinct polymeric film was observed on PPy_1_@P_0.5_‐CF_700_ compared to the unmodified CF_700_. This morphological change confirms the successful deposition of PPy onto P_1_‐CF_700_, consistent with our prior findings that phosphorus doping does not alter the morphology of pristine carbon materials [36]. Focused ion beam scanning electron microscopy (FIB‐SEM) cross‐sectional analysis further revealed a uniform PPy nanofilm with a thickness of approximately 80.2 nm on the carbon fibers, indicating effective and homogeneous coating of the P_1_‐CF_700_ surface (Figure 1c). Additionally, the N content in PPy_1_@P_0.5_‐CF_700_ increased sharply from 2.4% to 9.1% after PPy nanocoating (Figure 1d and Figure S1). Due to PPy belonging to a class of organic amine polymers, the increase of N content in PPy_1_@P_0.5_‐CF_700_ further confirmed the successful construction of PPy nanocoating on 3D CF.

The SSA of CF_700_ and PPy_1_@P_0.5_‐CF_700_ was determined to be 1.33 and 1.22 m^2^/g, respectively (Figure 1e). The comparable SSA values suggest that the PPy nanocoating strategy effectively preserves the porous architecture of the pristine carbon material, in contrast to conventional PPy/carbon composite synthesis methods which often lead to significant pore blockage [37, 38]. The pore size distribution (inset, Figure 1e) further confirms that both CF_700_ and PPy_1_@P_0.5_‐CF_700_ possess abundant macropores, with negligible porosity loss after PPy modification. These findings collectively demonstrate that the PPy nanocoating successfully maintains the intrinsic morphology and porosity of the 3D CF. The ζ potential measurements as a function of pH (Figure S2) reveal that the point of zero charge (pH_zpc_) shifted from 3.2 for CF_700_ to 8.8 for PPy_1_@P_0.5_‐CF_700_. The significant increase in pH_zpc_ indicates a reversal of the surface charge from negative to positive, underscoring the role of the PPy coating in enhancing the surface electric field. Such a modification is expected to favor the electrostatic attraction of anionic PFAS molecules.

**FIGURE 2:**
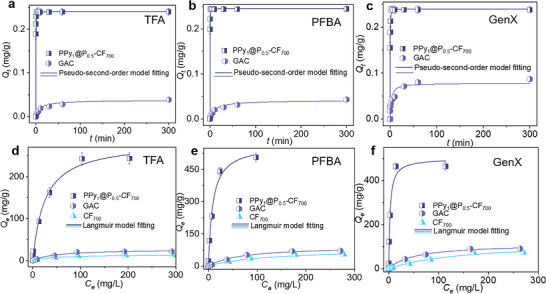
Nonlinear pseudo‐second‐order kinetic plot of GAC and PPy_1_@P_0.5_‐CF_700_ for the sorption of TFA (a), PFBA (b), and GenX (c); Sorption isotherms with Langmuir fitting on TFA (d), PFBA (e) and GenX (f) by CF_700_, GAC, and PPy_1_@P_0.5_‐CF_700_.

FTIR spectra of as‐synthesized materials are presented in Figure S3. A prominent peak at 1428 cm^−1^ in pure PPy is assigned to the C‐N stretching vibration of Py rings [39]. For P_0.5_‐CF_700_, the appearance of a peak at 1030 cm^−1^ corresponds to P = O/P‐O stretching, confirming successful P doping and the presence of P‐containing functional groups (PFGs) [40]. Notably, the coexistence of characteristic PPy and PFG peaks in PPy_1_@P_0.5_‐CF_700_ verifies the successful formation of PPy nanocoating on 3D CF. Additionally, XPS survey spectra (Figure S4) provide further evidence, showing distinct N and P signals in PPy_1_@P_0.5_‐CF_700_ that are absent in the individual P_0.5_‐CF_700_ and PPy reference materials. The elemental discrepancy confirms the successful construction of PPy nanocoating on 3D CF. Furthermore, the P 2p spectrum of Fe@P_0.5_‐CF_700_ (P_0.5_‐CF_700_ after Fe^3+^ adsorption) exhibited a new peak at 134.4 eV, attributed to Fe‐O‐P bonding [41]. Concurrently, the relative amounts of P‐O (50.9%) and P‐C (35.2%) decreased in comparison to pristine P_0.5_‐CF_700_ (53.8% and 46.2%; Figure 1f), indicating that PFGs participate in coordinating Fe^3+^. The presence of Fe 2p signals in Fe@P_0.5_‐CF_700_, and their subsequent disappearance in PPy_1_@P_0.5_‐CF_700_ (Figure 2d and Figure S1), suggest that Fe^3+^ was effectively confined by P‐doped CF (P_0.5_‐CF_700_) and consumed during the in situ oxidative polymerization of pyrrole, leading to the formation of a nanostructured PPy coating [42]. In summary, comprehensive morphological, structural, and chemical analyses confirm that the PPy nanocoating not only preserves the porous framework of CF but also significantly enhances its surface electric field. These synergistic modifications are anticipated to provide abundant and effective sorption sites for the subsequent capture of PFAS.

### 2.2 PFAS Sorption Performance

According to the optimized synthesis conditions, PPy_1_@P_0.5_‐CF_700_ exhibited the highest removal efficiency of PFBA in Milli‐Q water among all prepared sorbents, significantly outperforming materials lacking either PPy coating or P‐doping (e.g., PPy_1_@CF_700_ and P_0.5_‐CF_700_; Figure S6). These results underscore the critical role of the PPy nanocoating in enhancing PFAS sorption. As a proof of concept, the optimal PPy_1_@P_0.5_‐CF_700_ was selected for further investigation of PFAS sorption performance.

The sorption kinetics for ultrashort‐chain TFA, short‐chain PFBA, and emerging GenX were determined using PPy_1_@P_0.5_‐CF_700_ and compared with commercial GAC in Milli‐Q water. As shown in Figure S7a–c, PPy_1_@P_0.5_‐CF_700_ achieved efficient removal of >85% of TFA, PFBA, and GenX within 60 s, reaching sorption equilibrium in less than 300 s with PFAS removal exceeding 95%, demonstrating ultrafast and efficient PFAS removal. In contrast, the benchmark GAC exhibited considerably lower removal efficiencies (<35%) and required an equilibrium time of approximately 300 min under identical conditions. The estimated sorption rate constants (*k*_2_) of the three PFAS on PPy_1_@P_0.5_‐CF_700_ exceeded 3800 g/mg/h according to fitting results of the nonlinear pseudo‐second‐order model (Figure 2a–c and Table S1), representing a more than 21‐fold enhancement over GAC under the same conditions. These kinetic results indicated that the PPy nanocoating significantly improves the sorption capacity of 3D CF for hydrophilic PFAS, which are typically poorly removed by conventional carbon materials [43, 44]. Notably, few modified carbon materials have been reported to achieve such high *k*_2_ values, particularly for ultrashort‐chain TFA and emerging GenX. For instance, reported *k*_2_ values were only 2.65 g/mg/h for GAC [45], and 0.0324 g/mg/h for PPy/biochar composites [35] during PFBA sorption. Therefore, PPy_1_@P_0.5_‐CF_700_ reported in this study represents an efficient and novel carbon‐based material for rapid removal of ultrashort‐ and short‐chain PFAS as well as emerging PFAS alternatives in water.

Sorption isotherm was conducted to elucidate the binding mechanisms and maximum sorption capacities of PPy_1_@P_0.5_‐CF_700_, GAC, and CF_700_ for PFAS. As shown in Figure S8a–c, the equilibrium sorption capacities of PPy_1_@P_0.5_‐CF_700_ for TFA, PFBA, and GenX all exceeded 250 mg/g, representing at least an 8‐fold improvement over GAC and CF_700_ under identical conditions. The Langmuir constants (*K*_L_), indicative of binding affinity between PFAS and sorbents, were 0.0402–0.3801 L mg^−1^ for PPy_1_@P_0.5_‐CF_700_, compared to 0.0066–0.0218 L mg^−1^ for CF_700_ and 0.0147–0.0204 L mg^−1^ for GAC (Figure 2d–f and Table S2). The *K*_L_ values of PPy_1_@P_0.5_‐CF_700_ for tested PFAS were more than 2 times those of CF_700_ and GAC, indicating that ultrashort‐ and short‐chain PFAS and emerging alternatives to PFAS have a stronger binding affinity with PPy_1_@P_0.5_‐CF_700_ [46]. This further validates that the PPy nanocoating reverses the sorption affinity of CF toward hydrophilic PFAS. Furthermore, the maximum sorption capacities (*q*_m_) of PPy_1_@P_0.5_‐CF_700_ for TFA, PFBA, and GenX were calculated as 283.2, 572.1 and 510.2 mg g^−1^, respectively, which are at least 6.5 and 5.1 times higher than those of CF_700_ and GAC, respectively (Figure 2d–f and Table S2). Interestingly, the *q*_m_ of PPy_1_@P_0.5_‐CF_700_ for GenX (C6) was lower than that for PFBA (C4), contrary to the conventional trend where sorption capacity increases with perfluoroalkyl chain length due to enhanced solvophobicity and dispersion forces [47]. This suggests that the hydrophilic ether group (C‐O‐C) in GenX reduces its affinity for the sorbent, favoring its retention in the aqueous phase. The sorption capacities of PPy_1_@P_0.5_‐CF_700_ for the three PFAS were significantly higher than those of previously reported sorbents (Table S3). For instance, the *q*_m_ values of TFA were 5.88 and 6.45 times greater than those of quaternary nitrogen‐grafted GAC (32.9 mg g^−1^) [48], and surface‐defunctionalized activated CF (30 mg g^−1^) [49], respectively. Similarly, the *q*_m_ values of GenX were 2.32 and 2.55 times greater than those of magnetic fluorinated polymer sorbents (219 mg g^−1^) [25], and amine‐functionalized covalent organic frameworks (200 mg g^−1^) [50], respectively. Given the increasing environmental prevalence of ultrashort‐ and short‐chain PFAS and emerging PFAS due to the regulation and transformation of long‐chain analogues, the performance of PPy_1_@P_0.5_‐CF_700_ sets a new benchmark for PFAS sorbents. The PPy nanostructured coating strategy presents a promising approach for designing advanced 3D carbon materials with enhanced capability to remove diverse PFAS from water.

### 2.3 Breakthrough Experiments

**FIGURE 3:**
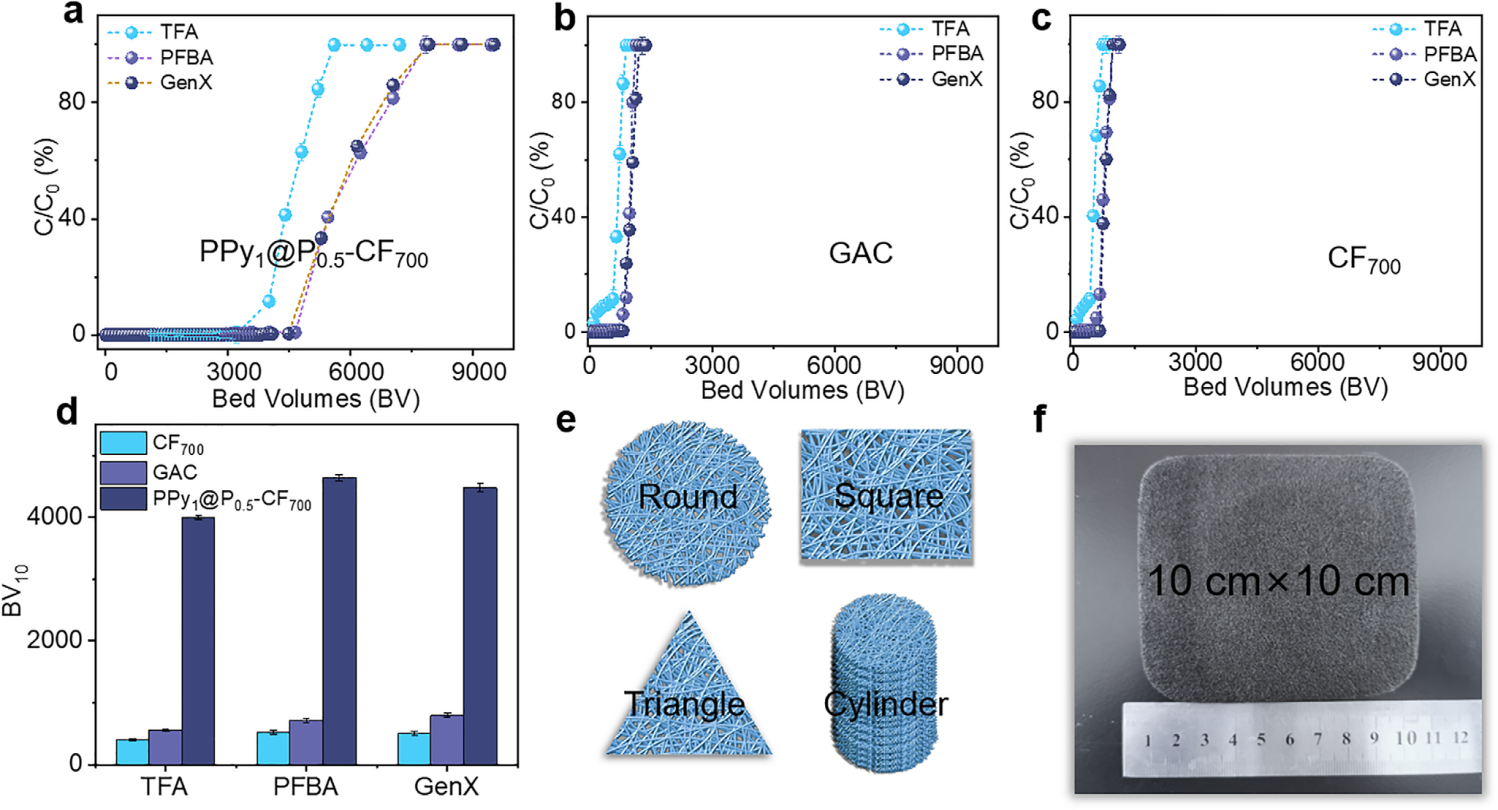
Breakthrough curves of three typical PFAS by the column packed with PPy_1_@P_0.5_‐CF_700_ (a), GAC (b) and CF_700_ (c); comparison of 10% breakthrough bed volumes of the three columns (d); scheme illustration of PPy@P‐CF with different shape (e); photograph of PPy_1_@P_0.5_‐CF_700_ with 10 cm × 10 cm × 0.2 cm size for treatment of 6 L water (f).

Encouraged by the exceptional performance of PPy_1_@P_0.5_‐CF_700_ in batch PFAS removal, we further evaluated its efficacy under dynamic flow conditions using a custom‐designed short column (Figure S9). The 10% breakthrough threshold (BV_10_), defined as the bed volume at which the effluent PFAS concentration exceeded the limit of quantification (Text S1), was used to evaluate the removal performance of sorbents in column experiments. As illustrated in Figure 3a, the PPy_1_@P_0.5_‐CF_700_ column exhibited prolonged breakthrough times and high BV_10_ values for all three representative PFAS. Consistent with previous findings [47], a chain‐length‐dependent breakthrough trend was observed, with the ultrashort‐chain TFA breaking through earliest, followed by GenX and then PFBA. Notably, although GenX (C6) possesses a longer perfluoroalkyl chain than PFBA (C4), its earlier breakthrough can be attributed to the hydrophilic ether oxygen (C‐O‐C) in its structure, which likely reduces its affinity for the sorbent compared to a purely perfluorinated chain of similar length. This chain‐length‐dependent trend was also evident in control experiments with CF_700_ and GAC (Figure 3b,c). However, both CF_700_ and GAC were significantly more prone to breakthrough than PPy_1_@P_0.5_‐CF_700_ under identical conditions. The BV_10_ values of the three PFAS ranged from 400 BV to 500 BV for CF_700_ column and from 560 BV to 800 BV for GAC column (Figure 3b,c). In stark contrast, the PPy_1_@P_0.5_‐CF_700_ column achieved BV_10_ values exceeding 4000 BV for all PFAS, representing an enhancement of at least 5‐fold compared to the CF_700_ and GAC columns (Figure 3d). Specifically, the BV_10_ of the most hydrophilic TFA in the PPy_1_@P_0.5_‐CF_700_ column was > 6 times higher than that in the CF_700_ and GAC columns. These dynamic test results highlighted the great potential of PPy_1_@P_0.5_‐CF_700_ for practical application in treating PFAS‐contaminated water and effectively addressing the universal mass transfer limitations often associated with the sorption of hydrophilic PFAS.

Moreover, the scalability and versatility of the PPy nanostructured coating strategy were demonstrated by extending the synthesis to fabricate bulk 3D PPy@P‐CF with dimensions on the centimeter scale and in various geometries (e.g., square, triangle and cylinder) (Figure 3e). These customizable forms allow the material to be adapted for use in diverse scenarios and locations, significantly complementing the available treatment options for ultrashort‐chain, short‐chain, and emerging PFAS. For instance, a large‐format PPy_1_@P_0.5_‐CF_700_ film measuring 10 cm × 10 cm × 0.2 cm was successfully fabricated (Figure 3f), which can be integrated into adsorption film devices for water treatment. These findings substantially extend the potential for large‐scale application of 3D PPy_1_@P_0.5_‐CF_700_ and highlight the general applicability of the interface engineering strengthening and pore preservation strategy for modifying 3D carbon materials.

### 2.4 Sorption Mechanisms

**FIGURE 4:**
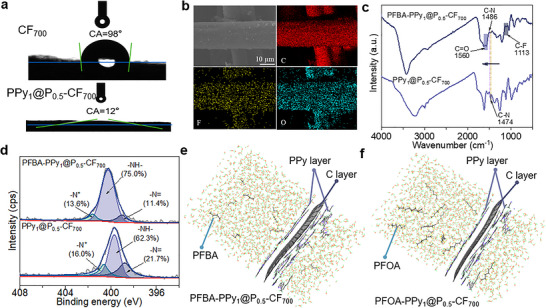
Water contact angle of CF_700_ and PPy_1_@P_0.5_‐CF_700_ (a); SEM image of PPy_1_@P_0.5_‐CF_700_ after PFBA sorption and corresponding F elemental EDS mappings (b); FTIR spectra of PPy_1_@P_0.5_‐CF_700_ before and after PFBA sorption (c); N1s XPS spectra of PPy_1_@P_0.5_‐CF_700_ before and after PFBA sorption (d); MDS of PFBA (e) and PFOA (f) sorption by PPy_1_@P_0.5_‐CF_700_.

To elucidate the adsorption mechanisms of PFAS on PPy_1_@P_0.5_‐CF_700_, a series of characterizations including water contact angle (WCA), EDS mapping, FTIR, and XPS were conducted before and after the adsorption of TFA, PFBA and GenX. As shown in Figure 4a, the WCA of pristine CF_700_ was 98°, indicating its hydrophobic nature, whereas the WCA of PPy_1_@P_0.5_‐CF_700_ significantly decreased to 12°, demonstrating that the PPy nanocoating effectively converted the surface property from hydrophobic to hydrophilic. This enhanced hydrophilicity improved the aqueous dispersibility of PPy_1_@P_0.5_‐CF_700_, thereby facilitating the mass transfer of PFAS molecules to the adsorption sites and contributing to the high sorption capacity [51]. The EDS mapping of PPy_1_@P_0.5_‐CF_700_ after PFAS sorption revealed a uniform distribution of F atoms (Figure 4b), primarily originating from the C‐F chains of the adsorbed PFAS. This observation suggests that PFAS molecules were effectively captured either on the surface of PPy_1_@P_0.5_‐CF_700_ or within the macropores formed by the interlaced CF via pore‐filling mechanisms [39, 51]. Furthermore, the FTIR spectra of PPy_1_@P_0.5_‐CF_700_ after PFAS sorption exhibited two new peaks at 1113 and 1560 cm^−1^ (Figure 4c), which are attributed to the C‐F stretching vibration and C = O stretching mode of PFAS molecules, respectively, confirming the successful adsorption of PFAS. Notably, a significant shift in the C‐N stretching vibration from 1474 to 1486 cm^−1^ was observed after PFAS loading, indicating strong electrostatic interactions between the carboxylate groups (COO^−^) of TFA, PFBA, and GenX and the positively charged nitrogen species (‐N^+^) in the PPy layer of PPy_1_@P_0.5_‐CF_700_ [35]. Given that amine functional groups (AFGs) are known to enhance PFAS adsorption [52], the N1s XPS spectra of PPy_1_@P_0.5_‐CF_700_ were analyzed before and after PFAS sorption. As shown in Figure 4d, the N1s spectrum of the pristine material (PPy_1_@P_0.5_‐CF_700_) was deconvoluted into three peaks at 399.6, 400.5, and 401.7 eV, corresponding to imine (= NH‐, 21.7%), amine (‐NH‐, 62.3%), and positively charged nitrogen (‐N^+^, 16.0%), respectively [37]. After PFAS adsorption, these peaks shifted to 399.8, 400.7, and 401.9 eV, respectively, and the relative contents changed to 11.4%, 75.0%, and 13.6%. Furthermore, these changes confirm the involvement of AFGs in PFAS sorption, with the ‐N^+^ sites playing a critical role in binding anionic PFAS via electrostatic interactions.

To gain further insight into the adsorption mechanisms at the molecular level, MDS calculation was conducted. As shown in Figure 4e, the PPy layer reduced the repulsive force caused by the hydrophobicity of the solid sorbent at the water‐solid interface, enhancing the wettability of PPy_1_@P_0.5_‐CF_700_ and electropositivity and facilitating the mass transfer of hydrophilic and electronegative PFBA molecules toward the adsorbent surface [15]. The MDS results also showed that PFBA adsorbed onto the PPy layer of PPy_1_@P_0.5_‐CF_700_ with its carboxylic head group interacting with AFGs and its C‐F chain tail oriented perpendicular to the surface, underscoring the importance of AFGs in PFAS binding. In contrast, for the hydrophobic CF_700_, the water‐solid interface heavily impeded the approach of PFBA, leading to its low adsorption capacity (Figure S10). To assess the generality of this mechanistic insight, we extended the MDS analysis to PFOA, a representative long‐chain PFAS. As shown in Figure 4f, a similar trend was observed for PFOA: while its head group also interacted with AFGs on PPy_1_@P_0.5_‐CF_700_, it tended to remain in the aqueous phase when interacting with unmodified CF_700_ (Figure S11). This consistent behavior across different PFAS confirms that the PPy nanolayer fundamentally alters the surface characteristics of CF_700_, favoring PFAS adsorption. It is worth noting that, unlike PFBA, the longer perfluoroalkyl chain of PFOA adopted a parallel orientation to the PPy surface (Figure 4f), which can be attributed to hydrophobic interactions between the chain and the adsorbent surface [22]. In conclusion, PPy_1_@P_0.5_‐CF_700_ exhibits high adsorption performance for PFAS irrespective of their C‐F chain length. The inherent hydrophilicity of short‐chain and emerging PFAS typically discourages their migration from the aqueous phase to hydrophobic adsorbent surfaces. However, the PPy nanocoating not only improves hydrophilicity of adsorbent surfaces to mitigate the mass transfer resistance at the water‐solid interface but also enhances the surface electric field, enabling rapid and efficient adsorption of these hydrophilic PFAS.

### 2.5 Photocatalytic Degradation of PFAS after Sorption and Mechanisms

**FIGURE 5:**
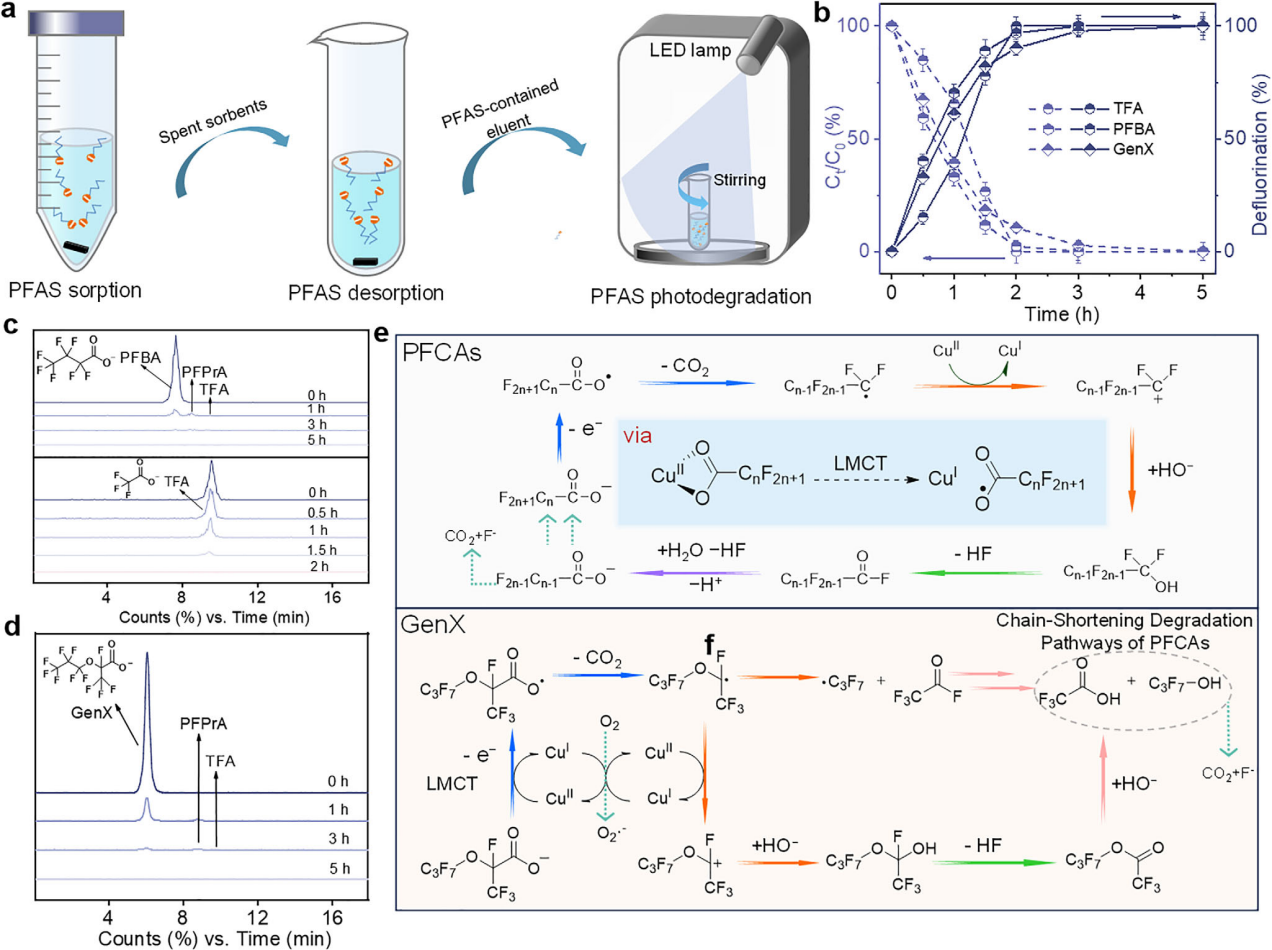
Developed PFAS photodegradation procedure (a); Degradation and defluorination rates of the single‐component TFA, PFBA and GenX (b); HPLC‐MS/MS chromatograms system of PFBA and TFA degradation (c) and GenX degradation (d); Proposed Chain‐Shortening Degradation Pathways of PFCAs and GenX (Blue: Decarboxylation, Yellow: Hydroxylation, Green: Elimination, Purple: Hydrolysis) (e).

The high‐performance sorption capacity of PPy@P‐CF achieves efficient PFAS concentration, yet it does not permanently remove PFAS. Additionally, desorbing PFAS for regeneration and reusability of PPy@P‐CF is also necessary to evaluate its practical applicability. Solvent washing, based on the “like dissolves like” principle, is a widely employed technique for regenerating spent sorbents [22]. However, this process generates organic waste solutions containing high concentrations of PFAS, posing a great challenge to further treatment. Herein, we developed a “Concentrate‐&‐Destroy” strategy including PFAS sorption, desorption and photodegradation in eluent to address these limits (Figure 5a). TFA, PFBA and GenX were selected as representative PFAS to evaluate the regeneration efficiency. Nearly 100% of the adsorbed PFAS could be eluted from the sorbent using a mixed solution of 0.1 mol/L NaOH water solution/acetonitrile (1:999, v:v) irrespective of the C‒F chain length. The regenerated sorbent maintained high PFAS removal efficiency (>95%) over at least five consecutive adsorption‐desorption cycles (Figure S12). The collected eluent (enrichment factor of 25) was subsequently irradiated with a 365 nm LED for 300 min, achieving complete degradation (100%) and defluorination (100%) of TFA, PFBA, and GenX (Figure 5b). Targeted analysis of degradation products identified TFA and perfluoropropionic acid (PFPrA) as intermediates during the degradation of PFBA (0–60 min) (Figure 5c) and GenX (up to 180 min) (Figure 5d), respectively. For the ultrashort‐chain TFA, no intermediates were detected within 120 min (Figure 5c). The complete disappearance of target PFAS and fluorinated intermediates in the high‐performance liquid chromatography coupled with tandem mass spectrometry (HPLC‐MS/MS) chromatograms by the end of the reaction confirmed the thorough degradation of all three PFAS (Figure 5c,d).

The experimental results indicate that the PFAS degradation mechanisms proceed via a LMCT process, wherein electron transfer from PFAS to Cu^2^^+^ initiates a chain‐shortening degradation pathway, accompanied by the reduction of Cu^2^^+^ to Cu^+^ [53, 54]. For perfluoroalkyl carboxylic acids (PFCAs), photoexcitation induces LMCT between Cu^2+^ and the perfluorocarboxylate anion (C_n_F_2n+1_COO^−^), leading to the formation of an acyl radical (C_n_F_2n+1_COO•), which subsequently undergoes decarboxylation. Using PFBA (C_3_F_7_COO^−^) as an example, LMCT results in the generation of C_3_F_7_COO•, which decarboxylates to yield the perfluoroalkyl radical C_3_F_7_• [54]. This radical is oxidized by Cu^2+^ to form a cationic intermediate, which reacts with OH^−^ to produce C_3_F_7_OH. Subsequent HF elimination forms C_2_F_5_COF, which hydrolyzes to yield C_2_F_5_COO^−^ (Figure S13) [29]. The product C_2_F_5_COO^−^ contains one less carbon than PFBA and undergoes further chain‐shortening and defluorination steps, ultimately mineralizing PFBA into CO_2_ and HF (Figure 5e). In addition to the chain‐shortening products (short‐chain PFCAs), no other possible fluorinated byproducts, such as H/F exchange products (for example, C_3_F_6_HCOO^−^ and C_3_F_5_H_2_COO^−^), were detected (Figure S14). Notably, under O_2_‐free (N_2_) conditions, PFBA defluorination was limited to only ≈1%, likely due to the absence of an external oxidant preventing the re‐oxidation of Cu^+^ to Cu^2+^, thereby halting the reaction cycle [54]. ESR measurements confirmed the involvement of molecular oxygen: a distinct DMPO‐superoxide (O_2_•^−^) adduct was detected in the presence of Cu^2^^+^ and deprotonated PFBA, whereas no such signal appeared in control experiments with Cu^2+^ alone (Figure S15). Thus, O_2_ facilitates the oxidation of Cu^+^ back to Cu^2+^, sustaining the catalytic cycle for continuous PFAS degradation.

For GenX, a representative perfluoroalkyl ether carboxylic acid (PFECA), degradation also begins with LMCT‐induced electron loss, followed by decarboxylation to form C_3_F_7_OCF(CF_3_)• [55]. The presence of an ether oxygen enables C‐O bond cleavage, producing CF_3_COF and C_3_F_7_• [32], both of which subsequently follow defluorination pathways analogous to those described for PFBA. Alternatively, C_3_F_7_OCF(CF_3_)• can form a perfluoroalkyl alcohol (C_3_F_7_OCF(CF_3_)OH) (Figure 5e), which undergoes HF elimination to yield a perfluoroalkyl ester (C_3_F_7_OC(CF_3_)OF). Hydrolysis of this ester initiates further defluorination via the same reaction pathway as PFBA (Figure 5e).

### 2.6 Application of Integrated Sorption Concentration and Photochemical Destruction of PFAS in Practical Contaminated Water

**FIGURE 6:**
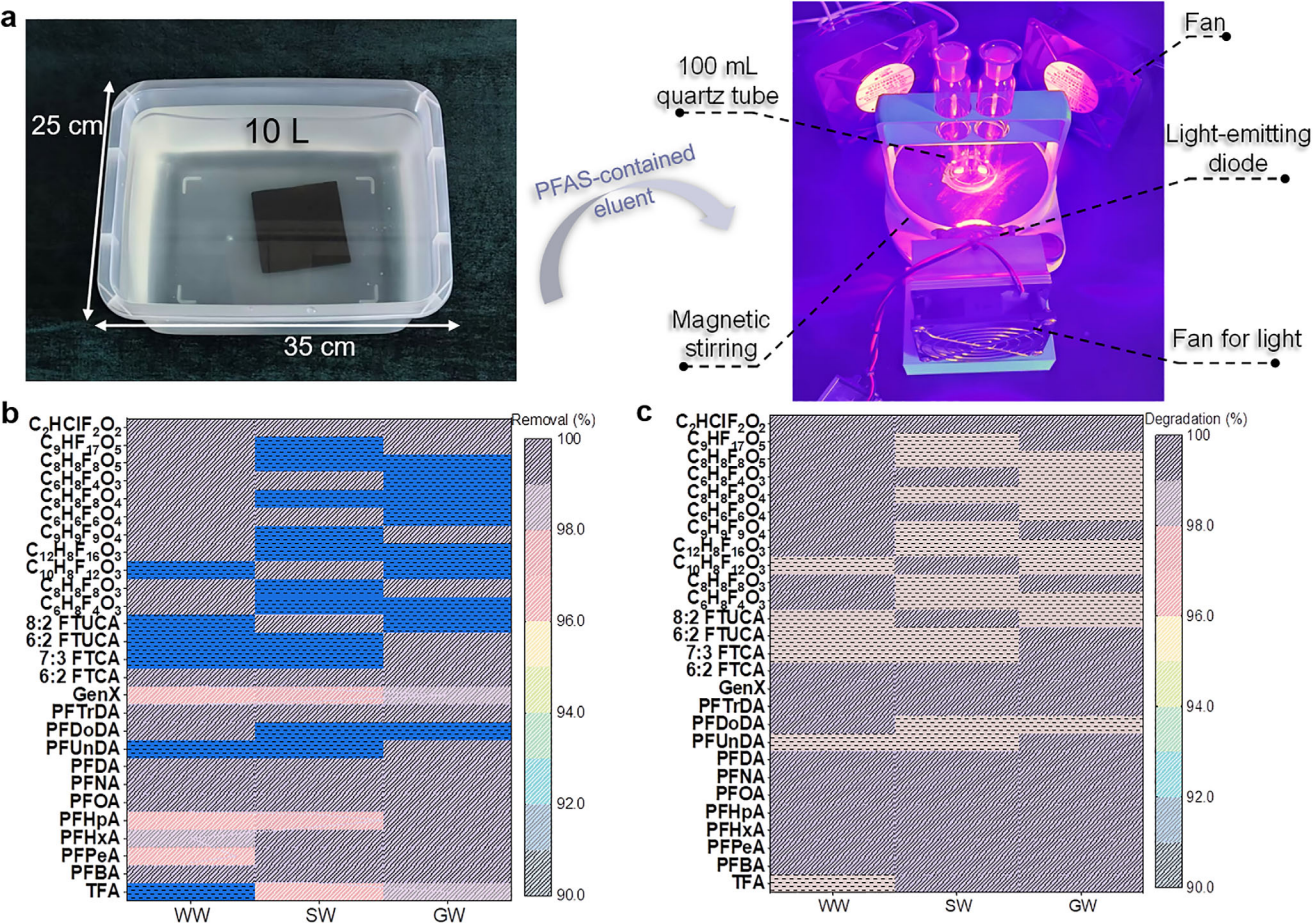
Photography of PFAS sorption by 10 cm × 10 cm × 0.2 cm PPy@P‐CF in 10 L SW and subsequent PFAS photodegradation by the 365 nm LED lamp irradiation (a); Removal efficiencies of 16 kinds of target PFAS and 11 nontarget analytes of emerging PFAS by PPy1@P0.5‐CF700 in three real PFAS‐contaminated water samples (Blue indicates no PFAS detected, original data are summarized in Table S6) (b); Degradation efficiencies to 16 kinds of target PFAS and 11 nontarget analysis emerging PFAS in the three real PFAS‐contaminated water after sorption (light gray indicates no PFAS detected) (c).

To evaluate the practical applicability of our developed material and “Concentrate‐&‐Destroy” strategy, the bulk 10 cm × 10 cm × 0.2 cm PPy_1_@P_0.5_‐CF_700_ was used to treat 10 L of three different actual contaminated water samples, wastewater (WW) (ΣPFAS = 115.95 µg L^−1^), surface water (SW) (ΣPFAS = 0.806 µg L^−1^), and groundwater (GW) (ΣPFAS = 2.01 µg L^−1^) (Figure 6a). These samples contained a diverse range of PFAS, including 11 perfluoroalkyl carboxylic acids (PFCAs), 1 PFECA, 2 fluorotelomer carboxylic acids (FTCAs), and 2 fluorotelomer unsaturated carboxylic acids (FTUCAs), as identified in our previous study (Table S4) [56]. Furthermore, nontarget analysis of these waters revealed the presence of 11 emerging PFAS (Table S5), including an ultrashort chlorinated PFCA (Cl‐PFCA, C2) [56]. Remarkably, PPy_1_@P_0.5_‐CF_700_ demonstrated highly efficient removal (> 96%) for all the detected PFAS across the three water matrices (Figure 6b). Specifically, the material achieved > 96% removal for the highly hydrophilic TFA, > 97% for the emerging GenX, and > 97% for PFBA. While the complex composition of WW and SW led to a slight reduction in the removal efficiency for certain hydrophilic PFAS compared to the less complex GW, the consistently high removal rates (>96%) in all real water samples underscore the broad‐spectrum PFAS removal capability of PPy_1_@P_0.5_‐CF_700_. This performance highlights its significant application potential and strong anti‐interference capacity in remediating complex, PFAS‐contaminated water environments.

Moreover, PPy_1_@P_0.5_‐CF_700_ achieved near‐quantitative removal (≈100%) of the 11 emerging PFAS identified via nontarget analysis in all three real water samples (Figure 6b and Table S6). To the best of our knowledge, few studies have combined target and nontarget analyses to comprehensively assess the removal of such newly identified PFAS. The outstanding sorption performance of PPy_1_@P_0.5_‐CF_700_ for both known and emerging PFAS underscores the effectiveness of the surface electric field strengthening and pore preservation strategy employed in this work, which is highly competitive, if not superior, to other modification methods reported for 3D carbon materials (Table S3).

The diverse constituents in real water, particularly dissolved organic and inorganic matter, can potentially inhibit UV‐based degradation of PFAS by competing for photons or scavenging reactive species [29]. Despite this challenge, our integrated approach successfully achieved complete photodegradation of all 27 PFAS (from both target and nontarget analyses) present in the real contaminated water eluents after a 5 h UV irradiation period (Figure 6c). This result demonstrates the broad‐spectrum degradation efficacy and strong practical application potential of the integrated interface engineering‐enhanced carbon felt sorption and photodegradation strategy. This “Concentrate‐&‐Destroy” technology effectively breaks the “forever cycle” of PFAS by overcoming the key limitations of conventional sorption technologies, namely, difficult regeneration and the failure to destroy PFAS. As a proof‐of‐concept, this study presents an integrated “sorption concentration‐photochemical destruction” strategy for remediating PFAS‐contaminated water. The approach leverages the high adsorption capacity and tunable morphology of the PPy@P‐CF sorbent, followed by a photodegradation step that ensures complete defluorination under environmentally benign and operationally mild conditions. Future work will evaluate the efficacy and robustness of the photodegradation step under realistic flow‐through configurations and across multiple operational cycles to rigorously assess the strategy's scalability, practical applicability, and long‐term sustainability.

## 3 Conclusions

This study demonstrates a highly efficient “Concentrate‐&‐Destroy” strategy for efficient concentration and complete destruction of ultrashort‐chain, short‐chain, and emerging PFAS from contaminated water, employing a 3D PPy@P‐CF integrated with a LMCT photochemical system. The engineered PPy_1_@P_0.5_‐CF_700_ features a uniform, electropositive polypyrrole nanocoating (≈80.2 nm) that significantly enhances the surface electric field while preserving the macroporous structure of the CF. This design enables ultrafast sorption kinetics (equilibrium < 300 s) and exceptional adsorption capacities, up to 283.2 mg g^−1^ for TFA, 572.1 mg g^−1^ for PFBA, and 510.2 mg g^−1^ for GenX, which are 5.1–6.5 times greater than those of GAC and unmodified CF. In dynamic column tests, the material exhibited outstanding performance with the BV_10_ exceeding 4000 BV for TFA, PFBA, and GenX, demonstrating strong potential for continuous treatment applications. Moreover, PPy@P‐CF achieved > 96% removal of 27 target and emerging PFAS from real contaminated water matrices, including wastewater, surface water, and groundwater. The material also showed excellent reusability over five sorption–regeneration cycles. Critically, the concentrated PFAS in the regeneration eluent were completely degraded and defluorinated within 5 h via a UV‐LED/Cu^2^^+^ photochemical process, breaking the perpetual PFAS cycle. Mechanistic studies revealed that the PPy layer improves interfacial mass transfer and facilitates electrostatic interactions, enabling efficient capture of hydrophilic PFAS. This integrated “concentrate‐and‐destroy” strategy not only overcomes the limitations of conventional adsorption technologies but also offers an adoptable solution for PFAS remediation in diverse water treatment scenarios, and offers guidance for the application of next‐generation functionalized 3D carbon materials in environmental applications.

## 4 Experimental Section

### 4.1 Chemicals and Materials

The commercial CF (thickness 0.2 cm; porosity 85%; ash content 150 ppm; density 0.55–0.60 g/cm^3^; tensile strength 100–160 MPa in lengthways and 20–25 MPa in laterally; heat stability 2750°C in inert gases and 380°C in air) was obtained from Shanghai Lishuo Composite Material Technology Co., Ltd, China. A full list of chemical reagents and materials is provided in Text S2. Single standard stock solutions of TFA, PFBA, and GenX were prepared in methanol at 1–50 g L^−1^. 16 target PFAS and their detailed information (e.g., suppliers and internal standards) are listed in Table S7. Unless otherwise specified, Milli‐Q water with a resistivity of 18.2 MΩ·cm was used during the experiments. Adsorption performance was benchmarked against a commonly reported and extensively used coconut‐shell GAC adsorbent (Shanghai Aladdin Biochemical Technology Co., Ltd., China; Text S2).

### 4.2 Adsorbents Preparation and Characterization

Phosphorus‐doped CF was synthesized by co‐pyrolyzing nonphosphorus biomass and KH_2_PO_4_ according to our previously reported method to confine Fe^3+^ for inducing the formation of PPy nanocoating [40], and it was denoted as P*_m_*‐CF*_n_*, where *m* represents the different mass ratios of KH_2_PO_4_ and pristine CF (0, 0.5, 1, and 2), and *n* represents the pyrolysis temperature (500–900°C). The PPy@P‐CF composite (1.0 cm × 1.0 cm × 0.2 cm) was synthesized by a novel *in‐situ* “Fe^3+^‐confined” oxidative polymerization method (Figure 1a) and denoted as PPy*_x_* @P*_m_*‐CF*_n_* (*x* represents the additive volume of the Py monomer (0, 0.5, 1.0, and 2.0 mL) during synthesis). The detailed preparation procedures for P‐CF and PPy@P‐CF are provided in Text S3.

The morphology, structure, and chemical composition of the as‐prepared materials were characterized by scanning electron microscopy (SEM) equipped with energy dispersion spectroscopy (EDS), FIB‐SEM, automated chemisorption analysis, X‐ray photoelectron spectroscopy (XPS), Fourier transform infrared (FTIR) spectroscopy, and zeta potential analysis (Text S4).

### 4.3 PFAS Sorption Experiments

The batch adsorption experiments were carried out by exposing 150 mg of PPy@P‐CF to 50 mL of water solution in 50 mL polypropylene centrifuge tubes on an oscillator at 180 rpm at 25°C. After adding the sorbents and deionized water to the tubes, the sorption system was pre‐equilibrated for 2 h, after which the designated amount of PFAS stock solution was spiked (methanol content ≤ 0.1%). The pH of the sorption system was 6.5 ± 0.1 and was not adjusted. After sorption, a 1.0 mL suspension was collected and filtered through a 0.22 µm polyethersulfone membrane for subsequent concentration analysis using HPLC‐MS/MS. For the single‐component sorption kinetic experiment, the initial concentrations of TFA, PFBA, and GenX are 100 µg L^−1^ which represented the environmental‐related concentrations near PFAS‐contaminated source points, and at each predetermined time (0–48 h), the suspension was sampled for HPLC‐MS/MS analysis. For the sorption isotherm experiment, the relatively high PFAS concentrations ranging from 1 to 500 mg L^−1^ were adopted to examine the maximum sorption capacity of PPy@P‐CF as far as possible, and the suspensions were oscillated for 24 h to reach equilibrium. Then, 1.0 mL of the suspension was sampled and diluted according to the calibration curve range before HPLC‐MS/MS analysis. The breakthrough experiments were conducted on the homemade PFAS trapping column (inner diameter = 12 mm) (details about the columns and pretreatment are shown in Text S5). PFAS‐spiked (5.0 µg L^−1^ for each PFAS) water was pumped into the column at a flow rate of 3.0 mL min^−1^, and 1.0 mL of the filtrate was collected at predetermined intervals, and stored at 4°C for HPLC‐MS/MS analysis. Instrumental analysis, quality control, and data analysis are described in detail in Text S1.

### 4.4 MDS

To obtain more insights into the mechanisms through which the adsorption of short‐chain PFAS is enhanced by the PPy nanocoating, MDS calculation was used to simulate this process. The graphene sheets were used to mimic the hydrophobic CF surface. PFOA, PFBA and PPy were parameterized with Gaussian 09 at the B3LYP level with the 6–31G(d,p) basis set to generate the underlying electrostatic potential maps of each molecule. A larger supercell with a size of 32 Å × 38 Å × 59 Å was used to perform sorption experiments and was constructed by replicating the primitive unit cell. All details about MDS calculation are shown in Text S6.

### 4.5 Photochemical Destruction of PFAS in Eluent After Sorbent Regeneration

The sorbents after single‐solute PFAS (100 µg L^−1^) sorption were easily picked out through a clean forceps and eluted by 2 mL of a 0.1 mol L^−1^ NaOH water solution/acetonitrile (1:999, v:v) to regenerate (Text S7). Five sorption‐desorption cycles were conducted to investigate the regeneration and reusability of PPy@P‐CF. For the photochemical destruction of PFAS after sorbent regeneration, the 1 µmol trifluoromethanesulfonate (Cu[OTf]_2_) was added to the eluent, and the solution with highly concentrated PFAS (2500 µg L^−1^) was exposed to a 365 nm light‐emitting diode lamp for 0–300 min to degrade PFAS (Text S7). The PFAS degradation product, radical and F^−^ concentration were identified by ultrahigh‐performance liquid chromatography‐high‐resolution mass spectrometry (UPLC‐HRMS), electron spin resonance (ESR) spectroscopy and a portable multimeter connecting an ion‐selective electrode, respectively. The reaction pathway was deduced according to the above analysis results. Details about analytical methods were shown in Text S8.

### 4.6 Application of Integrated Sorption Concentration and Photochemical Destruction of PFAS in Practical Contaminated water

Three practical PFAS‐contaminated water samples, namely, WW, SW, and GW, were collected from near a fluorochemical industrial park polluted with various target and nontarget PFAS [56]. The locations and water quality parameters (e.g., TOC, pH, Cl^−^, NO_3_^−^, SO_4_^2−^, Na^+^, Mg^2+^, Ca^2+^) are all described in detail in Table S8. 16 target PFAS and 11 nontarget PFAS in those water bodies were detected in our previous report [56], and the pretreatment, initial concentrations of target PFAS, and semi‐quantified concentrations of nontarget PFAS are described in detail in Tables S4 and S5. For the sorption experiment in real PFAS‐contaminated water, 15 g of PPy@P‐CF (10 cm × 10 cm × 0.2 cm) and 10 L of practical water samples were used, and the other procedures were the same as the above batch experiments. After PFAS sorption, the sorbents were picked out, and solid‐phase extraction was performed to concentrate the practical water according to our previous method [57], followed by LC‐MS/MS and ultrahigh‐performance liquid chromatography‐high‐resolution mass spectrometry (UHPLC‐HRMS) analysis. The spent sorbents were eluted by 50 mL of a 0.1 mol L^−1^ NaOH water solution/acetonitrile (1:999, v:v) and 2 µmol Cu[OTf]_2_ was added. PFAS in the eluent were degraded by photochemical destruction for 300 min, and the detailed experiments were described as above and in Text S7.

## Author Contributions

**Hongwen Sun**: validation, writing – review and editing, funding acquisition. **Hao Yu**: conceptualization, investigation, writing – original draft, writing – review and editing, data curation. **Hongyi Li**: validation, writing – review and editing. **Peng Zhang**: methodology, supervision, writing – review and editing, funding acquisition, validation. **Jialei Guo**: validation, writing – review and editing, investigation. **Feng He**: validation, writing – review and editing.

## Conflicts of Interest

The authors declare no conflicts of interest.

## Supporting information




**Supporting File**: advs76133‐sup‐0001‐SuppMat.docx.

## Data Availability

The data that support the findings of this study are available from the corresponding author upon reasonable request.
