# Supplementary material for: Efficient Concentration and Complete Destruction of Short‐Chain and Emerging PFAS in Contaminated Water via Integrated Interface Engineering‐Enhanced Carbon Felt Sorption and Photochemical Processes
Source: Adv Sci (Weinh). 2026 Jun 15:e76133. Online ahead of print. doi: 10.1002/advs.76133 (PMC13336416; doi:10.1002/advs.76133)
Supplement: Supplementary file 1 — Supporting File: advs76133‐sup‐0001‐SuppMat.docx. [file ADVS-9999-e76133-s001.docx]

**Supplementary Information**

**Efficient Concentration and Complete Destruction of Short-Chain and Emerging PFAS in Contaminated Water via Integrated Interface Engineering-Enhanced Carbon Felt sorption and Photochemical Processes**

*Hao Yu ^1^, Jialei Guo ^1^, Peng Zhang,** *Hongyi Li, Feng He, Hongwen Sun*

H. Yu

MOE Key Laboratory of Pollution Processes and Environmental Criteria, College of Environmental Science and Engineering, Nankai University, Tianjin 300350, China

School of Petrochemical Engineering & Environment, Zhejiang Ocean University, Zhoushan 316000, China

J. Guo, P. Zhang and H. Sun

MOE Key Laboratory of Pollution Processes and Environmental Criteria, College of Environmental Science and Engineering, Nankai University, Tianjin 300350, China

Haihe Laboratory of Sustainable Chemical Transformations, Tianjin, 300192, China

H. Li and F. He

Institute of Environmental Processes and Pollution Control, and School of Environment and Ecology, Jiangnan University, Wuxi 214122, China

^1^ Both authors contributed equally to this work

* Corresponding authors: Peng Zhang

Nankai University

38 Tongyan Road, Jinnan District, Tianjin 300350, China

Phone: 86-22-23509241

E-mail: nkzhangpeng@nankai.edu.cn

42 Pages

8 Text

8 Tables

15 Figures

**Tables of Content**

| Text | Page | Content |
| --- | --- | --- |
| S1 | 4 | Instrumental analysis of PFAS, quality control, and statistical analysis |
| S2 | 7 | Chemicals and materials |
| S3 | 8 | Preparation of adsorbents |
| S4 | 10 | Characterization |
| S5 | 11 | Column Preparation |
| S6 | 12 | Molecular Dynamics Simulation (MDS) |
| S7 | 13 | Regeneration and PFAS degradation |
| S8 | 15 | Identification for PFAS photochemical destruction |

| Tables | Page | Content |
| --- | --- | --- |
| S1 | 17 | Sorption kinetic parameters |
| S2 | 18 | Sorption isotherm parameters |
| S3 | 19 | Comparison of PFAS sorption on different carbon materials and AERs |
| S4 | 20 | iLOQ, method detection limits (MDL), and concentrations of target PFAS in the three real PFAS-contaminated water |
| S5 | 21 | PFAS obtained by suspect target and nontarget screening and semi-quantified concentrations |
| S6 | 22 | PFAS removal in the three real PFAS-contaminated water |
| S7 | 23 | Details on the target analysis of 16 PFAS and internal standards in UPLC-MS/MS |
| S8 | 25 | Locations and water quality parameters of the three real PFAS-contaminated water |

| Figures | Page | Content |
| --- | --- | --- |
| S1 | 26 | SEM images and EDS mapping of pristine CF_700_ |
| S2 | 27 | ζ potential of pristine CF_700_ and PPy_1_@P_0.5_-CF_700_ at different pH values |
| S3 | 28 | FTIR spectra of PPy, P_0.5_-CF_700_ and PPy_1_@P_0.5_-CF_700_ |
| S4 | 29 | XPS spectra of PPy, P_0.5_-CF_700_, and PPy_1_@P_0.5_-CF_700_ |
| S5 | 30 | XPS spectra of P_0.5_-CF_700_, Fe@P_0.5_-CF_700_, and PPy_1_@P_0.5_-CF_700_ |
| S6 | 31 | Optimization of preparation conditions (pyrolysis temperature (500 ℃, 700 ℃, 900 ℃), KH_2_PO_4_:CF (m:m) (0, 0.5, 1, 2), Volume of Py added (0, 0.5, 1, and 2 mL)) |
| S7 | 32 | Sorption kinetics for TFA (a), PFBA (b), GenX (c) on GAC and PPy_1_@P_0.5_-CF_700_ |
| S8 | 33 | Sorption isotherms for TFA (d), PFBA (e), GenX (f) on CF_700_, GAC, and PPy_1_@P_0.5_-CF_700_ |
| S9 | 34 | Photograph of a homemade short sorption column |
| S10 | 35 | MDS of PFBA sorption by CF_700_ |
| S11 | 36 | MDS of PFOA sorption by CF_700_ |
| S12 | 37 | Regeneration ability of PPy_1_@P_0.5_-CF_700_ (single-solute system, [PFAS]_0_= 100 µg/L) |
| S13 | 38 | Chromatograms and MS/MS spectrum of [C_2_F_5_COO]^-^ (m/z=162.9824) (Degradation time: 60 min; mass tolerance: 5 ppm) |
| S14 | 39 | Searched for chain-shortening and H/F exchange fluorinated intermediates (Degradation time: 60 min; mass tolerance: 5 ppm) |
| S15 | 40 | ESR spectra of DMPO-O_2_•^−^ |

**Text S1 Instrumental analysis of PFAS, quality control, and statistical analysis**

Separation of ultrashort-chain TFA (C2) was achieved using an ion-exchange RSpak JJ-50 2D column (2.0 mm i.d. × 150 mm length, 5 μm; Shodex, Japan). The mobile phase composition was 20% of 50 mM ammonium acetate (at pH 9) and 80% of methanol (v/v), at a flow rate of 0.25 mL/min under isocratic conditions. The column temperature was set at 30 ℃ and the injection volume at 10 µL. For short- and long-chain PFAS (C4-C13) and emerging PFAS, the Athena C18-WP column (50 mm × 2.1 mm, i.d. 3 μm, ANPEL, Shanghai, China) was used. The binary mobile phases were water containing 2.5 mM ammonium acetate (A) and methanol containing 2.5 mM ammonium acetate (B). The gradient elution mode was as follows: 0-0.5 min = 94% A; 0.5-5.5 min = 94% A to 5% A; 5.5-8 min = 5% A; and 8-13 min = 94% A. The flow rate was set at 0.3 mL/min and the injection volume was 10 μL. Analysis of PFASs was performed with a high-performance liquid chromatograph-tandem mass spectrometer (HPLC-MS/MS). The instrument was an Agilent 1260 series liquid chromatography interfaced with an Agilent G6460C triple quadrupole mass spectrometer (Agilent Technologies, USA) operated in the electrospray negative ionization multiple-reaction monitoring (MRM) mode. The multiple-reaction monitoring (MRM) mode was used for measurement. Curtain gas (CUR), collision activated dissociation gas (CAD), heater gas GS1 and GS2 were set at 35 psi, 8.00 psi, 45.00 and 40.00 psi, respectively, and turbo ion spray voltage was -4500 V. The instrument parameters for the quantification of each compound by HPLC-MS/MS are shown in Table S1.

For quality control, all accessible polytetrafluorethylene materials were avoided to minimize background contamination. Polypropylene materials were selected during the whole experiment. The procedure blanks and blank spiked samples were used as quality controls, which was the same procedure as the samples. The Milli-Q water (as blank) was always detected with every batch of samples. The loss caused by the adsorption of PFASs on the walls of PP tubes and filter media was less than 2%, which was confirmed by control experiments. Prior to analysis, 5 ng of each isotopically labeled internal standard was added and mixed with 0.3 mL of samples in the polypropylene vial to correct instrument signal drift as well as matrix effects. The instrumental limits of detection (iLODs) and limit of quantification (iLOQs) were defined as the peak of analyte that yielded a signal-to-noise (S/N) ratio of 3 and 10, respectively. In this study, the iLODs for TFA, PFBA, and GenX were 96.1 ng/L, 13.3 ng/L, and 68.3 ng/L, respectively. The iLOQs for TFA, PFBA, and GenX were 560.1 ng/L, 38.1 ng/L, and 275.1 ng/L, respectively. The internal standard calibration curve comprised samples with incremental levels of native standards (0.05, 0.10, 0.50, 2, 5, 10, 20, 50, 100 µg/L, the starting point of the curve determined by iLOQs of the individual PFAS), prepared in methanol (prior to instrumental analysis) and spiked with 5 ng internal standard. The calibration curves showed suitably determined coefficients for all target analytes (*R*^2^ >0.99).

Although internal standards were added prior to extraction, exact matching internal standards could not be used for some compounds. A potential pitfall was noted regarding the IS correction of TFA. Since ^13^C_2_-labelled TFA was not available at the time of quantification, ^13^C_1_- labeled TFA was used. The signal of 1-fold labeled TFA could be impacted by natural isotopic overlap in native TFA. The artifact could significantly affect TFA quantification for those highly contaminated samples (underestimation of TFA concentration due to the unwanted signal increase of ^13^C_1_-TFA). Therefore, in this study, the use of ^13^C_1_-TFA in concentration correction was avoided and correction with the ^13^C_4_-labelled PFBA internal standard was applied.

All experiments were run in triplicate, and the means and standard deviations of results were used in statistical analysis. Statistical significance was determined by a one-tailed t-test. The *p*-values were determined using *T*-values and degrees of freedom, and the results were regarded as statistically significant when *p* < 0.05.

The following equations were used to calculate the sorption efficiency (% removal) and sorption capacity (*Q*) of PFAS on synthesized sorbents:

$\%Removal = \frac{(C_{0}-C_{e})}{C_{0}}$ (1)

$Q = (\frac{C_{0}-C_{e}}{m})V$ (2)

Where 𝐶_0_ and 𝐶_𝑒_ are the initial and equilibrium PFAS concentrations (mg/L) in an aqueous solution, respectively; 𝑚 is the added sorbent mass (g); and 𝑉 is the volume of solution (L) in the sorption system.

The pseudo-second-order model is given as follows: $\frac{t}{q_{t}}=\frac{1}{k_{2}Q_{e}^{2}}+\frac{t}{Q_{e}}$ (3)

Where *Q*_e_ and *q*_t_ are the amounts of PFAS adsorbed on the adsorbent (mg/g) at equilibrium and at time *t*, respectively, and *k*_2_ is the rate constant of pseudo-second-order adsorption (g/mg/h).

The Langmuir model is given as follows: $Q_{e}=K_{L}Q_{m}C_{e}/(1+K_{L}C_{e})$ (4)

where *K_L_* (L/mg) is the Langmuir sorption constant and where *Q*_m_ is the maximum sorption capacity (mg/g).

**Text S2 Chemicals and materials**

The physical properties of GAC used during the experiments are shown below: particle size: 10-20 mesh; BET surface area: 739 m^2^/g; BET surface area distribution: < 2 nm (607 m^2^/g), 2-50 nm (123 m^2^/g), >50 nm (9 m^2^/g); Total pore volume: 0.38 cm^3^/g; Pore volume distribution: < 2 nm (0.31 cm^3^/g), 2-50 nm (0.07 cm^3^/g), > 50 nm (0.00 cm^3^/g); iodine number: 775 mg/g. Pyrrole monomer was purchased from Macklin Biochemical Company (Shanghai, China) and stored at 4 ℃ before use. All other chemicals and reagents are commercially available and used as received unless otherwise stated.

**Text S3 Preparation of sorbents**

**Synthesis of P-CF**: First, 5 g of 1 cm × 1 cm × 0.2 cm CF and 5 g of KH_2_PO_4_ (P precursors; w:w = 1:1) were mixed with 200 mL of deionized water in a glass beaker and stirred at 500 rpm for 4 h at room temperature. Then, the mixture was freeze-dried in a vacuum freeze dryer. Different mass ratios of KH_2_PO_4_ and 1 cm × 1 cm × 0.2 cm CF of 0, 0.5, 1.0 and 2.0 were also designed. The obtained dried solid mixture was placed in an atmosphere-controlled furnace with an N_2_ atmosphere and then pyrolyzed at 500, 700 and 900 ℃ for 0.5 h, respectively. Subsequently, 1 cm × 1 cm × 0.2 cm P-CF was obtained after the furnace naturally cooled to room temperature. The yielded P-CF samples were washed with deionized water until no phosphorus was detected in the solution, and then filtered and further freeze-dried, labeled as P*_m_*-BC*_n_*, with m representing the additive mass ratio of P precursor (0, 0.5, 1, and 2) and n representing the pyrolysis temperature (500, 700 and 900 ℃). Pristine 1 cm × 1 cm × 0.2 cm CF with a pyrolysis temperature of 700 ℃ was also prepared according to the above steps but was fabricated without the addition of P-containing compounds.

**Synthesis of PPy@P-CF composite**: The PPy@P-CF composite was synthesized by an innovative method. Firstly, the 1 cm × 1 cm × 0.2 cm P*_m_*-BC*_n_* was immersed in a 0.1 g/L FeCl_3_ solution (P-CF: FeCl_3_, m:m=1:2) following shaking for 12 h. The excess FeCl_3_ ensures the full contact between P*_m_*-CF*_n_* and it. The obtained mixture was dried by a vacuum freeze dryer. Then, the Fe^3+^-immersed P*_m_*-CF*_n_* (Fe@P*_m_*-CF*_n_*) was filtered via vacuum filtration using a Buchner funnel apparatus and washed with 10 mL DI water for 3 times to wash off the free Fe^3+^. The residual Fe^3+^ in washings can be collected and treated with evaporation and concentration for further utilization. Then, the Fe@P*_m_*-CF*_n_* was added to 50 mL aqueous solution containing different volume of pyrrole (Py) (0, 0.5, 1, and 2 mL), and the solution was stirred for 30 min at ambient temperature to well disperse Py. Finally, 15 mL of 6 M HCl solution was slowly added with a flow rate of 0.5 mL/min to continuously generate Fe^3+^ and the mixture was stirred for 24 h for Py monomer *in situ* polymerization. The final black products were collected and washed with ultrapure water and methanol until the filtrate was colorless and its pH was neutral. After drying at 60 °C in a vacuum oven for 10 h, the PPy coating P-CF composite was obtained and defined as PPy*_x_*@P*_m_*-CF*_n_* (*x* representing the additive volume of Py monomer). The 10 cm × 10 cm × 0.2 cm PPy*_x_*@P*_m_*-CF*_n_* was also synthesized by the same procedure except using the 10 cm × 10 cm × 0.2 cm CF.

**Text S4 Characterization**

The morphology was observed by scanning electron microscopy (SEM, ZEISS GeminiSEM 500). The focused ion beam scanning electron microscopy (FIB-SEM, ZEISS Crossbeam 540) was employed to collect 3D volumetric information and reveal the PPy-coating thickness of PPy_1_@P_0.5_-CF_700_. Following were the specific procedures of FIB-SEM operation: 1) automatic alignment, 2) clipping to the region of interest, and 3) denoising using a non-local means filter. The functional groups on the surface of the prepared materials were detected by Fourier transform infrared spectrometry (FTIR, NICOLET 5700, USA) in the wave number range of 400-4000 cm^-1^ in the transmission mode. The specific surface area and pore size distribution of the prepared materials were analyzed using Brunauer-Emmett-Teller (BET, ASAP 2460, USA). X-ray photoelectron spectroscopy (XPS, Thermo Scientific Escalab 250Xi, UK) analysis was conducted to identify the chemical composition of the prepared materials. The Zeta potentials of the as-prepared sorbents were determined using the Zeta Potential Analyzer (Malvern, UK).

**Text S5 Column Preparation**

150 mg of GAC, CF_700_ and PPy_1_@P_0.5_-CF_700_ were slowly added into the empty SPE column with the bottom frit while methanol was used to wet them. Then, the top frit was tightly packed and the obtained column volumes of GAC column, CF_700_ column, and PPy_1_@P_0.5_-CF_700_ column were 0.15 mL, 0.18 mL, and 0.20 mL, respectively. Before use, the column was purified with methanol (20 mL) and then water (20 mL).

**Text S6 Molecular Dynamics Simulation (MDS)**

An initial graphite structure unit cell was obtained from the Crystallography Open Database. All simulations were run with GROMACS 2022.6. Initial configurations were energy minimized with the method of the conjugated gradient until the maximum force on any atom was less than 100 kJ mol^−1^ nm^−1^. For the sorption simulations, a short equilibration was then run for 10 ns in the isothermal/isobaric ensemble (NPT) at 298.15 K and 1 atm with a timestep of 1 fs. Temperature was controlled with the V-rescale thermostat with a coupling constant set at 0.2 ps, while pressure was held constant with the C-rescale barostat and a 2 ps coupling constant. Upon completion of this short equilibration of 10 ns, a production run was initiated for 50 ns. Production runs were executed at the same 298.15 K and 1 atm but utilized a V-rescale thermostat and C-rescale barostat, both with coupling parameters set to 0.2 ps and 1 ps. Twelve PFAS molecules were randomly inserted into the simulation box and solvated with SPC/E water. Restrained electrostatic potential (RESP) method was utilized as implemented in the antechamber module of amber along with the generalized amber force field (GAFF) to derive the charge and geometric parameters for our MDS. Visualizations were generated with VMD software.

**Text S7 Regeneration and PFAS degradation**

For adsorption experiments, a piece of sorbent was exposed to 50 mL of water solution in 50 mL polypropylene centrifuge tubes on an oscillator at 180 rpm at 25 ℃. After adding the sorbents and deionized water to the tubes, the sorption system was pre-equilibrated for 2 h, after which the designated amount of single-solute PFAS stock solution was spiked to make the initial concentrations of single TFA, PFBA, and GenX be 100 µg/L. After adsorption, the PFAS-loaded sorbents were separated by a tweezer before drying at 60 ℃ in an oven and then immersed into 2 mL of a 0.1 mol/L NaOH water solution/acetonitrile (1:999, v:v), then it was shaken at 180 rpm for 2 h for desorption. The regenerated sorbents were rinsed with deionized water three times to wash out residual NaOH before subsequent usage. The PFAS concentrations in the regenerated solution (eluent) were analysed by HPLC-MS/MS. In addition, the reusability was also performed by five sorption-desorption cycles wherein sorption and desorption experiments were conducted as described above.

After regeneration, 1 µmol trifluoromethanesulfonate (Cu[OTf]_2_) was added to the eluent. Batch photochemical degradation of PFAS in filtrates was conducted in a photochemical reactor equipped with a 365 nm light-emitting diode lamp (LED, 50 W) and a quartz tube, and maintained at 21 ± 1 °C using a wind cooling system. The reaction mixture was magnetically stirred at a rate of 500 rpm. For the dark control groups, the quartz tube was completely covered with aluminum foil to prevent light exposure. Aliquots of 300 µL were withdrawn at predetermined time intervals and stored at 4 °C for subsequent analysis. Each experiment was performed in triplicate to ensure data accuracy. The PFAS and fluoride ion (F^−^) concentrations were analysed to determine the PFAS degradation rate. The concentration of F^−^ was determined using an ion-selective electrode (ISE, HACH, U.S.) connected with an HQ30D Portable Multi Meter (HACH, U.S.). The detection limit for F^−^ was 0.01 mg/L. The concentration of F^–^ was further verified by ion chromatography (Thermo Scientific, ICS5000+).

The defluorination rate ($ⅆ_{F^{-}}$) was calculated using eq. 5:

$$ⅆ_{F^{-}}=\frac{C_{F^{-}}}{C_{0}\times n} (5)$$

Where $C_{F^{-}}$ was the concentration of fluoride ion (µg/L); $C_{0}$ was the initial concentration of PFAS (µg/L); and n corresponded to the number of fluorine atoms in one PFAS molecule.

**Text S8 Identification for PFAS photochemical destruction**

The degradation intermediates of TFA, PFBA and GenX were identified using ultrahigh-performance liquid chromatography (UPLC; Ultimate 3000 Series, Thermo Fisher Scientific, Bremen, Germany) coupled with high-resolution Orbitrap mass spectrometry (HRMS, Thermo Fisher Scientific, Bremen, Germany). Analyte separation was achieved using a Syncronis C18 column (1.7 µm, 2.1 × 100 mm, Thermo, USA) thermostated at 40 °C; the mobile phase flow rate was 0.40 mL/min. The chromatographic gradient program was shown as follows:

| Time (min) | %A  Milli-Q water contained 2.5 mM ammonium acetate | %B  Acetonitrile contained 2.5 mM ammonium acetate |
| --- | --- | --- |
| 0 | 10 | 90 |
| 4 | 42 | 58 |
| 12.5 | 100 | 0 |
| 15.5 | 100 | 0 |
| 15.6 | 10 | 90 |
| 21.5 | 10 | 90 |

The heated electrospray ionization source was used with the following settings: sheath gas flow rate was 40 arbitrary units (a.u.), auxiliary gas flow rate 15 a.u., sweep gas flow rate 0 a.u., capillary temperature 200°C and vaporizer temperature 250°C. Spray voltage for negative ion was -2.5 kV (fast polarity-switching mode).

Electron spin resonance (ESR) spectroscopy was employed to investigate potential reactive species in the reaction systems. Measurements were conducted using a JES-FA200 spectrometer (JEOL) with the following parameters: sweep width of 100 G, microwave frequency of 9.22 GHz, modulation frequency of 100 kHz, modulation amplitude of 1.0 G, and microwave power of 1 mW. A 100 mM solution of 5,5-dimethyl-1-pyrroline N-oxide (DMPO) was utilized as the spin-trapping agent for O_2_•^−^. ESR measurements for O_2_•^−^ were conducted in a mixture of 10% DMSO and 90% MeCN. The samples were analyzed at 0, 5, and 10 min after irradiation with a 365 nm LED lamp.

**Table S1.** Sorption kinetic parameters.

| Adsorbates | PPy_1_@P_0.5_-CF_700_ | | GAC | | *k*_2_ (PPy_1_@P_0.5_-CF_700_)/*k*_2_ (GAC) |
| --- | --- | --- | --- | --- | --- |
|  | *k*_2_ (g/mg/h) | *R*^2^ | *k*_2_ (g/mg/h) | *R*^2^ |  |
| TFA | 5088 | 0.9985 | 154 | 0.9733 | 33 |
| PFBA | 6036 | 0.9981 | 129 | 0.9724 | 47 |
| GenX | 3836 | 0.9949 | 179 | 0.8806 | 21 |

**Table S2.** Sorption isotherm parameters.

|  | PPy_1_@P_0.5_-CF_700_ | | | GAC | | | CF_700_ | | | *Q*_m_ (PPy_1_@P_0.5_-CF_700_)/*Q*_m_ (GAC) | *Q*_m_ (PPy_1_@P_0.5_-CF_700_)/*Q*_m_ (CF_700_) |
| --- | --- | --- | --- | --- | --- | --- | --- | --- | --- | --- | --- |
| Adsorbates | *K*_L_ (L/mg) | *Q*_m_ (mg/g) | *R*^2^ | *K*_L_ (L/mg) | *Q*_m_ (mg/g) | *R*^2^ | *K*_L_ (L/mg) | *Q*_m_ (mg/g) | *R*^2^ |  |  |
| TFA | 0.0402 | 283.2 | 0.9938 | 0.0204 | 28.7 | 0.9823 | 0.0218 | 20.1 | 0.9927 | 9.8 | 14.0 |
| PFBA | 0.1150 | 572.1 | 0.9927 | 0.0147 | 91.8 | 0.9949 | 0.0080 | 81.3 | 0.9928 | 6.2 | 7.0 |
| GenX | 0.3801 | 510.2 | 0.9901 | 0.0189 | 100.2 | 0.9973 | 0.0066 | 78.4 | 0.9875 | 5.1 | 6.5 |

**Table S3.** Comparison of PFAS sorption on different materials.

| **Adsorbents** | **Acronym** | **PFAS**^a^ | **Capacity (mg/g)** | **Equilibrium time (h)** | **Ref.** |
| --- | --- | --- | --- | --- | --- |
| Quaternary nitrogen-grafted granular activated carbon | AWNQ | TFA | 32.9 | 24 | ^[1]^ |
| Surface defunctionalized activated carbon felt | DeACF | TFA | 30.0 | 48 | ^[2]^ |
| Bamboo-derived granular active carbon | Bamboo-derived GAC | PFOA | 494 | 24 | ^[3]^ |
| Magnetic activated carbon | MAC | PFOA | 408 | 2.0 | ^[4]^ |
| Pinewood-derived biochar pyrolysed at 700 ℃  Hardwood-derived biochar pyrolysed at 900 ℃ | PWC  HWC | PFBA | - | ~24  >72 | ^[5]^ |
| Reed stalk pyrolysed at 900 ℃ | RESCA-900 | PFBA | 6.82 | 24 | ^[6]^ |
| Waste wood biochar pyrolysed at 700 ℃ | - | PFBA | 10.2 | 144 | ^[7]^ |
| Pine spruce wood biochar pyrolysed at 800 ℃ | - | PFBA  PFOA | 2.00×10^-3^  0.165 | - | ^[8]^ |
| Anion exchange resins (IRA910) | AER (IRA910) | PFBA  PFOA | 639  1442 | 24  72 | ^[9]^ |
| Anion exchange resins (AI400)  Powder activated carbon  Granular activated carbon | AER (AI400)  PAC  GAC | PFOA | 1206  428  152 | >168  4  >168 | ^[10]^ |
| Magnetic fluorinated polymer sorbents | P2-9+@IONPs | GenX | 219 | 0.05 | ^[11]^ |
| Amine-functionalized covalent organic frameworks | 28%[NH_2_]–COF | GenX | 200 | ~0.5 | ^[12]^ |
| Fluoropolymer sorbent | PFPE-IEX+ | PFBA  GenX | 524  ~875 | ~1 | ^[13]^ |
| Trifluoroacetic acid functionalized Zr-MOFs | TFA-MOF-808 | PFBA | 311 | - | ^[14]^ |
| Hollow covalent organic framework | Hollow Cys-COF | PFBA | 246 | 0.08 | ^[15]^ |
| Cationic covalent organic framework | BT-BDB-COF | GenX | 680 | 12 | ^[16]^ |
| 3D Polypyrrole-coated carbon felt composite | 3D PPy_1_@P_0.5_-CF_700_ | GenX  PFBA  TFA | 510.2  572.1  283.2 | 0.08  0.08  0.08 | **This study** |

^a^: Mainly focusing on TFA, PFBA and GenX.

**Table S4.** iLOQ, method detection limits (MDL), and concentrations of target PFAS in the three real PFAS-contaminated water.

| PFAS | iLOQ (ng/L) | MDL (ng/L) | PFAS concentrations (ng/L)^[17]^ | | |
| --- | --- | --- | --- | --- | --- |
|  |  |  | Wastewater | Surface water | Groundwater |
| TFA | 560.1 | 0.70 | 13.6 | n.d. | n.d. |
| PFBA | 38.1 | 0.04 | n.d. | 6.7 | 77.8 |
| PFPeA | 27.4 | 0.02 | 201.0 | 16.8 | 54.7 |
| PFHxA | 32.3 | 0.04 | 90250.0 | 154.0 | 702.0 |
| PFHpA | 18.6 | 0.02 | 210.0 | 12.0 | 31.7 |
| PFOA | 35.1 | 0.01 | 24625.0 | 314.0 | 704.0 |
| PFNA | 16.4 | 0.02 | 128.0 | 5.3 | 16.3 |
| PFDA | 3.77 | 0.01 | 81.3 | 4.0 | 11.7 |
| PFUnDA | 7.98 | 0.01 | 114.0 | 2.8 | 6.3 |
| PFDoDA | 6.05 | 0.01 | n.d. | n.d. | 1.3 |
| PFTrDA | 9.54 | 0.01 | 10.7 | n.d. | n.d. |
| 7:3 FTCA | 3.11 | 0.01 | n.d. | n.d. | 4.4 |
| 6:2 FTCA | 95.6 | 0.08 | 38.2 | 184.0 | 271.0 |
| 6:2 FTUCA | 10.1 | 0.01 | n.d. | n.d. | 4.9 |
| 8:2 FTUCA | 3.66 | 0.01 | n.d. | 1.7 | n.d. |
| GenX | 0.52 | 0.01 | 96.8 | 45.4 | 91.2 |

Note: n.d. means not detected.

**Table S5.** PFAS obtained by suspect target and nontarget screening and semi-quantified concentrations.^[17]^

| PFAS molecular  Formula | Theoretical mass [M-H] | Observed mass [M-H] | Observed RT (min) | Confidence level | Target match | PFAS concentrations (ng/L) | | |
| --- | --- | --- | --- | --- | --- | --- | --- | --- |
|  |  |  |  |  |  | Wastewater | Surface water | Groundwater |
| C_6_H_8_F_4_O_3_ | 203.03368 | 203.03363 | 1.80 | 3 | PFHxA | 8.2 | n.d. | n.d. |
| C_8_H_8_F_8_O_3_ | 303.02729 | 303.02600 | 4.10 | 3 | PFOA | 13.1 | n.d. | 12.2 |
| C_10_H_8_F_12_O_3_ | 403.02090 | 403.02090 | 6.72 | 3 | PFDA | n.d. | 6.7 | n.d. |
| C_12_H_8_F_16_O_3_ | 503.01451 | 503.01492 | 8.77 | 3 | PFDoDA | 6.2 | n.d. | n.d. |
| C_9_H_9_F_9_O_4_ | 351.02843 | 351.02838 | 8.37 | 3 | PFNA | 4.2 | n.d. | 13.4 |
| C_6_H_6_F_6_O_4_ | 255.00975 | 255.00896 | 6.10 | 3 | PFHxA | 3.3 | 2.1 | n.d. |
| C_8_H_8_F_8_O_4_ | 319.02220 | 319.02222 | 8.17 | 3 | PFOA | 4.2 | n.d. | n.d. |
| C_6_H_8_F_4_O_3_ | 203.03368 | 203.03360 | 1.80 | 3 | PFHxA | 43.5 | 34.2 | n.d. |
| C_8_H_8_F_8_O_5_ | 335.01712 | 335.01770 | 9.04 | 2 | PFOA | 66.8 | n.d. | n.d. |
| C_9_HF_17_O_5_ | 510.94797 | 510.94775 | 11.11 | 3 | PFNA | 3.5 | n.d. | 2.7 |
| C_2_HClF_2_O_2_ | 128.95603 | 128.95503 | 1.15 | 2 | PFBA | 24.5 | 16.2 | 6.2 |

Note: n.d. means not detected.

**Table S6**. PFAS removal in the three real PFAS-contaminated water.

| **Class** | **PFAS** | **PFAS Removal (%)** | | |
| --- | --- | --- | --- | --- |
|  |  | **Wastewater** | **Surface water** | **Groundwater** |
| PFCAs | TFA | 96.13±0.27 | - | - |
|  | PFBA | - | 96.43±0.45 | 98.17±0.88 |
|  | PFPeA | 100.13±0.46 | 100.08±0.37 | 100.21±0.76 |
|  | PFHxA | 96.91±0.47 | 100.01±0.49 | 100.00±1.39 |
|  | PFHpA | 98.04±0.71 | 100.01±0.69 | 100.12±1.10 |
|  | PFOA | 96.04±0.67 | 97.50±0.40 | 99.31±1.02 |
|  | PFNA | 100.05±0.62 | 100.16±0.57 | 100.00±0.43 |
|  | PFDA | 100.13±0.80 | 100.12±1.21 | 100.22±1.14 |
|  | PFUnDA | 100.32±0.71 | 100.10±1.02 | 100.17±1.18 |
|  | PFDoDA | - | - | 100.02±0.74 |
|  | PFTrDA | 100.03±0.29 | - | - |
| PFECAs | GenX | 96.31±0.39 | 97.42±0.33 | 98.22±0.41 |
| FTCAs | 6:2 FTCA | 100.03±0.68 | 100.15±0.74 | 100.09±0.56 |
|  | 7:3 FTCA | - | - | 100.21±1.13 |
| FTUCAs | 6:2 FTUCA | - | - | 100.04±1.08 |
|  | 8:2 FTUCA | - | 100.00±0.92 | - |
| Class 1 | C_6_H_8_F_4_O_3_ | 100.00±0.78 | - | - |
|  | C_8_H_8_F_8_O_3_ | 100.02±1.03 | - | 100.04±0.36 |
|  | C_10_H_8_F_12_O_3_ | - | 100.06±1.05 | - |
|  | C_12_H_8_F_16_O_3_ | 100.12±0.97 | - | - |
| Class 2 | C_9_H_9_F_9_O_4_ | 100.23±1.02 | - | 100.12±0.63 |
| Class 3 | C_6_H_6_F_6_O_4_ | 100.06±0.67 | 100.00±0.73 | - |
|  | C_8_H_8_F_8_O_4_ | 100.17±0.83 | - | - |
| Class 4 | C_6_H_8_F_4_O_3_ | 100.21±1.17 | 100.09±0.78 | - |
|  | C_8_H_8_F_8_O_5_ | 100.18±1.09 | - | - |
| Class 5 | C_9_HF_17_O_5_ | 100.08±1.01 | - | 100.11±087 |
| Class 6 | C_2_HClF_2_O_2_ | 99.22±0.78 | 100.11±0.88 | 100.03±0.76 |

**Table S7**. Details on the target analysis of 16 PFAS and internal standards in UPLC-MS/MS.

| **Acronym** | **Name** | | **Supplier** | **[M-H]^-^ or [M+H]^+^** | **Precursor ion** | **Production ion** | **Internal standard** |
| --- | --- | --- | --- | --- | --- | --- | --- |
| TFA | Trifluoroacetic acid | | Wellington | C_2_F_3_O_2_^-^ | 113 | 69 | ^13^C-TFA/^13^C_4_-PFBA^a^ |
| PFBA | Perfluorobutanoic acid | | Wellington | C_4_F_7_O_2_^-^ | 213 | 169 | ^13^C_4_-PFBA |
| PFPeA | Perfluoropentanoic acid | | Wellington | C_5_F_9_O_2_^-^ | 263 | 219 | ^13^C_3_-PFPeA |
| PFHxA | Perfluorohexanoic acid | | Wellington | C_6_F_11_O_2_^-^ | 313 | 269 | ^13^C_2_-PFHxA |
| PFHpA | Perfluoroheptanoic acid | | Wellington | C_7_F_13_O_2_^-^ | 363 | 319 | ^13^C_4_-PFHpA |
| PFOA | Perfluorooctanoic acid | | Wellington | C_8_F_15_O_2_^-^ | 413 | 369 | ^13^C_4_-PFOA |
| PFNA | Perfluorononanoic acid | | Wellington | C_9_F_17_O_2_^-^ | 463 | 419 | ^13^C_5_-PFNA |
| PFDA | Perfluorodecanoic acid | | Wellington | C_10_F_19_O_2_^-^ | 513 | 469 | ^13^C_2_-PFDA |
| PFUnDA | Perfluoroundecanoic acid | | Wellington | C_11_F_21_O_2_^-^ | 563 | 519 | ^13^C_2_-PFUnDA |
| PFDoDA | Perfluorododecanoic acid | | Wellington | C_12_F_23_O_2_^-^ | 613 | 569 | ^13^C_2_-PFDoDA |
| PFTrDA | Perfluorotridecanoic acid | | Wellington | C_13_F_25_O_2_^-^ | 663 | 619 | ^13^C_2_-PFDoDA |
| 6:2 FTUCA | 6:2 fluorotelomer unsaturated acid | | Wellington | C_8_F_12_HO_2_^-^ | 357 | 293 | ^13^C_4_-PFOA |
| 8:2 FTUCA | 8:2 fluorotelomer unsaturated acid | | Wellington | C_10_F_16_HO_2_^-^ | 457 | 393 | ^13^C_4_-PFOA |
| 6:2 FTCA | 6:2 Fluorotelomer carboxylic acid | | Wellington | C_8_F_13_H_2_O_2_^-^ | 377 | 293 | ^13^C_4_-PFOA |
| 7:3 FTCA | 7:3 Fluorotelomer carboxylic acid | | Wellington | C_10_F_15_H_4_O_2_^-^ | 441 | 317 | ^13^C_4_-PFOA |
| GenX | Perfluoro-2-propoxypropanoic acid | | Wellington | C_6_F_11_O_3_^-^ | 285 | 169 | ^13^C_3_-GenX |
| **Internal standards** | |  |  |  |  |  |  |
| ^13^C-TFA | Trifluoro-1-[^13^C]acetic acid | | Wellington | ^13^C_4_F_7_O_2_^-^ | 114 | 69 |  |
| ^13^C_4_-PFBA | Perfluoro-n-[^13^C_4_]butanoic acid | | Wellington | ^13^C_4_F_7_O_2_^-^ | 217 | 172 |  |
| ^13^C_3_-PFPeA | Perfluoro-n-[3,4,5-^13^C_3_]pentanoic acid | | Wellington | ^12^C_2_^13^C_3_F_9_O_2_^-^ | 266 | 222 |  |
| ^13^C_2_-PFHxA | Perfluoro-n-[1,2-^13^C_2_]hexanoic acid | | Wellington | ^12^C_4_^13^C_2_F_11_O_2_^-^ | 315 | 270 |  |
| ^13^C_4_-PFHpA | Perfluoro-n-[1,2,3,4-^13^C_4_]heptanoic acid | | Wellington | ^12^C_3_^13^C_4_F_13_O_2_^-^ | 367 | 322 |  |
| ^13^C_4_-PFOA | Perfluoro-n-[1,2,3,4-^13^C_4_]-octanoic acid | | Wellington | ^12^C_4_^13^C_4_F_15_O_2_^-^ | 417 | 372 |  |
| ^13^C_5_-PFNA | Perfluoro-n-[1,2,3,4,5-^13^C_5_]-nonanoic acid | | Wellington | ^12^C_4_^13^C_5_F_17_O_2_^-^ | 468 | 423 |  |
| ^13^C_2_-PFDA | Perfluoro-n-[1,2-^13^C_2_]-decanoic acid | | Wellington | ^12^C_8_^13^C_2_F_19_O_2_^-^ | 515 | 470 |  |
| ^13^C_2_-PFUnDA | Perfluoro-n-[1,2-^13^C_2_]-undecanoic acid | | Wellington | ^12^C_9_^13^C_2_F_21_O_2_^-^ | 565 | 520 |  |
| ^13^C_2_-PFDoDA | Perfluoro-n-[1,2-^13^C_2_]-dodecanoic acid | | Wellington | ^12^C_10_^13^C_2_F_23_O_2_^-^ | 615 | 570 |  |
| ^13^C_3_-GenX | 2,3,3,3-Tetrafluoro-2-(1,1,2,2,3,3,3-heptafluoropropoxy)- ^13^C_3_-propanoic acid | | Wellington | ^12^C_3_F_11_^13^C_3_O_3_^-^ | 287 | 169 |  |

a: ^13^C_4_-PFBA was used to correct the concentrations of TFA.

**Table S8.** Locations and water quality parameters of the three real PFAS-contaminated water.

|  | **Waste water** | **Surface water** | **Groundwater** |
| --- | --- | --- | --- |
| Latitude °N | 31.788131 | 31.792060 | 31.775584 |
| Longitude °E | 120.803503 | 120.801050 | 120.800485 |
| pH | 5.2 | 7.3 | 7.6 |
| TOC (mg/L) | 23.0 | 6.3 | 1.8 |
| CO_3_^2-^ (mg/L) | 12.0 | 5.0 | 1.1 |
| Cl^-^ (mg/L) | 12.1 | 8.3 | 3.6 |
| SO_4_^2-^ (mg/L) | 126.0 | 42.0 | 54.0 |
| PO_4_^3-^ (mg/L) | 2.7 | 5.6 | 1.7 |
| NO_3_^-^ (mg/L) | 3.1 | 1.2 | 0.9 |
| Na^+^ (mg/L) | 75.3 | 23.4 | 20.6 |
| Mg^2+^ (mg/L) | 8.2 | 2.1 | 1.8 |
| Ca^2+^ (mg/L) | 9.1 | 3.2 | 2.9 |

**Figure S1.** SEM images and EDS mapping of pristine CF_700_.


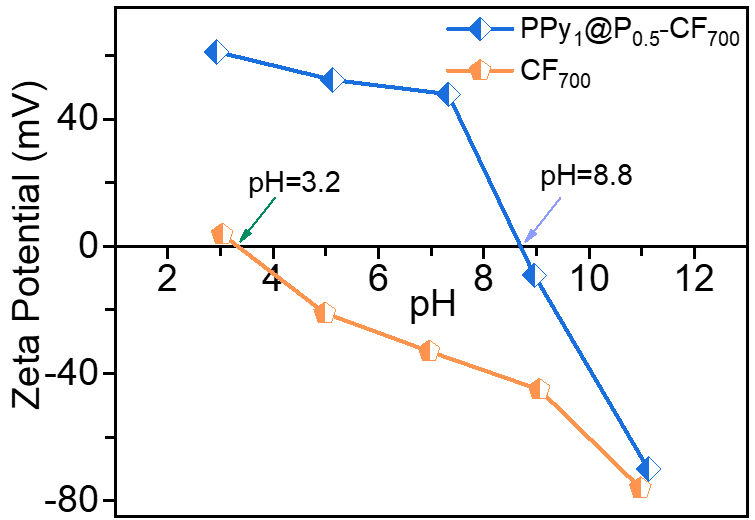


**Figure S2.** ζ potential of pristine CF_700_ and PPy_1_@P_0.5_-CF_700_ at different pH values.


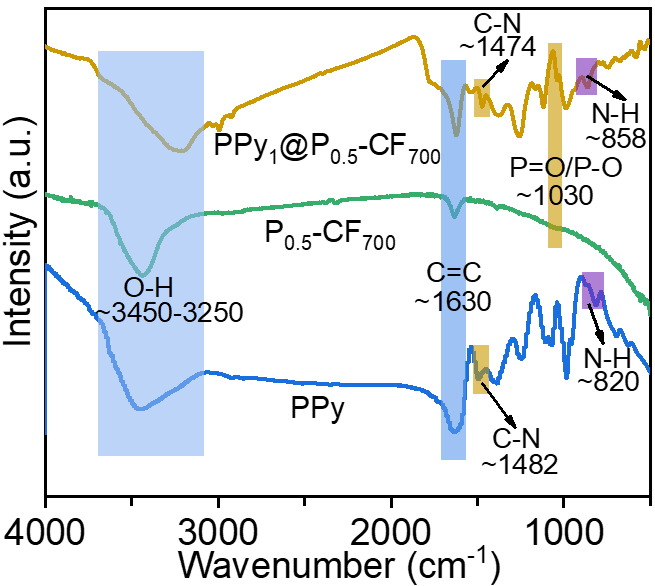


**Figure S3.** FTIR spectra of PPy, P_0.5_-CF_700_ and PPy_1_@P_0.5_-CF_700_.


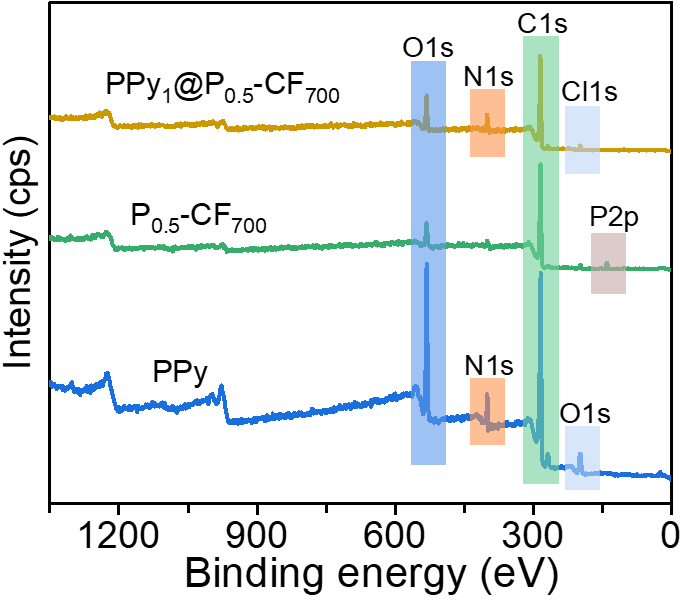


**Figure S4.** XPS spectra of PPy, P_0.5_-CF_700_, and PPy_1_@P_0.5_-CF_700_.


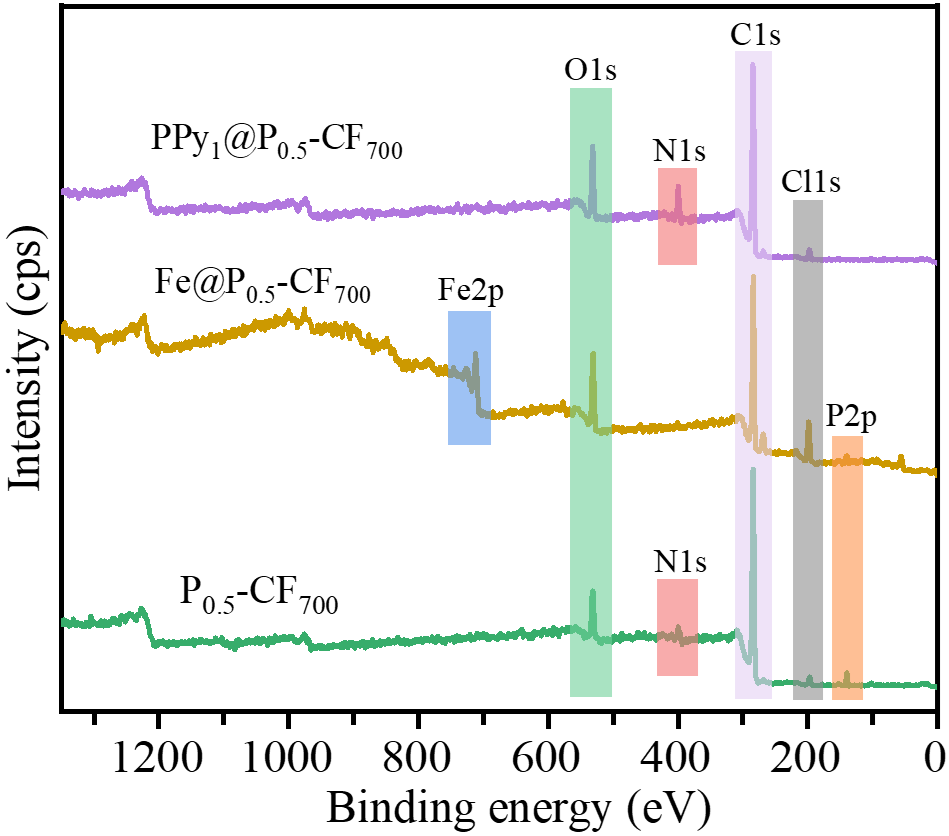


**Figure S5.** XPS spectra of P_0.5_-CF_700_, Fe@P_0.5_-CF_700_, and PPy_1_@P_0.5_-CF_700_.


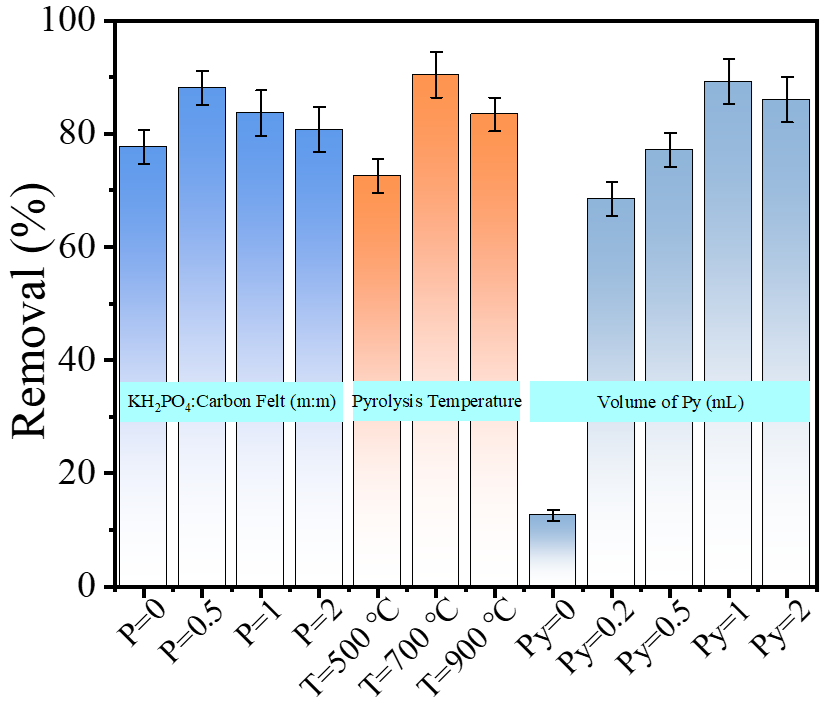


**Figure S6**. Optimization of preparation conditions (pyrolysis temperature (500 ℃, 700 ℃, 900 ℃), KH_2_PO_4_:CF (m:m) (0, 0.5, 1, 2), Volume of Py added (0, 0.5, 1, and 2 mL)). ([PFBA]= 20 mg/L, 1 cm × 1 cm × 1cm 3D PPy@P-CF (1 ± 0.1 g), equlibirum time: 24 h)


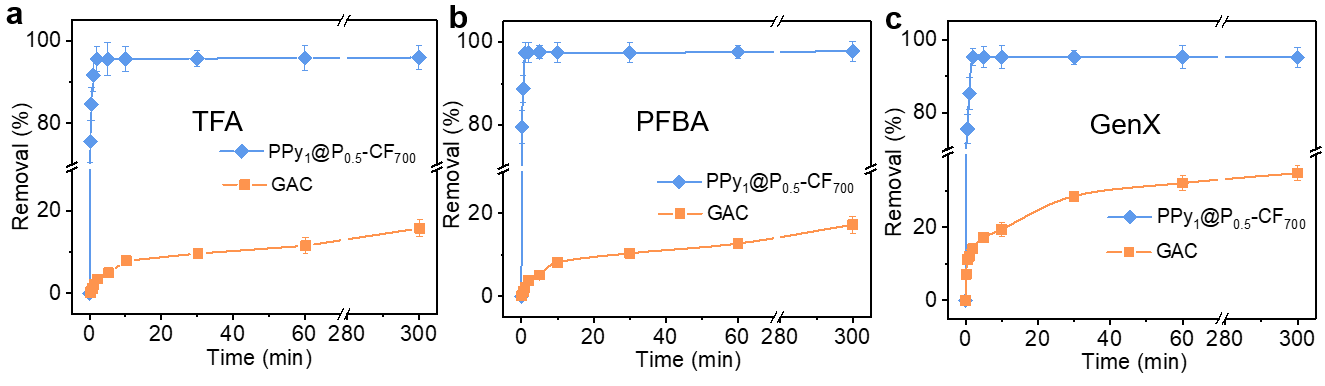


**Figure S7.** Sorption kinetics for TFA (a), PFBA (b), GenX (c) on GAC and PPy_1_@P_0.5_-CF_700_.


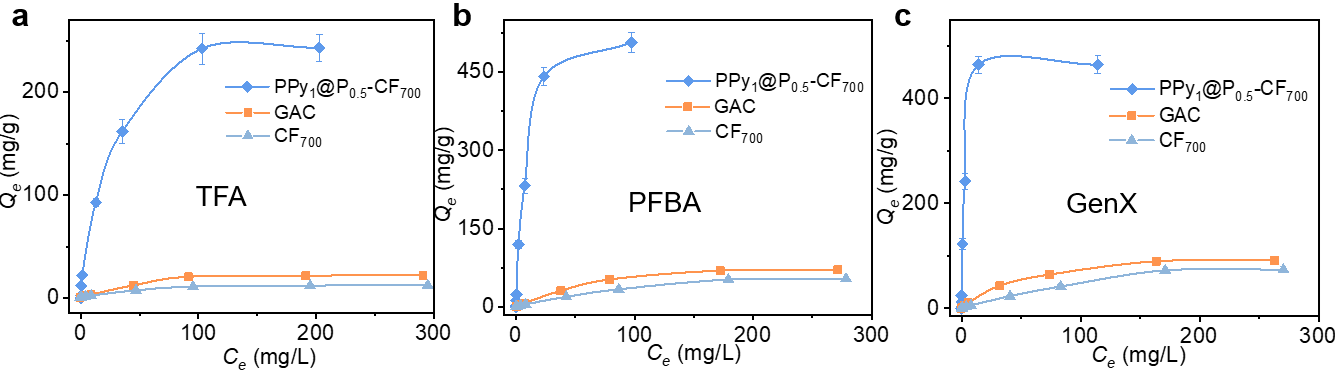


**Figure S8.** Sorption isotherms for TFA (a), PFBA (b), GenX (c) on CF_700_, GAC, and PPy_1_@P_0.5_-CF_700_.


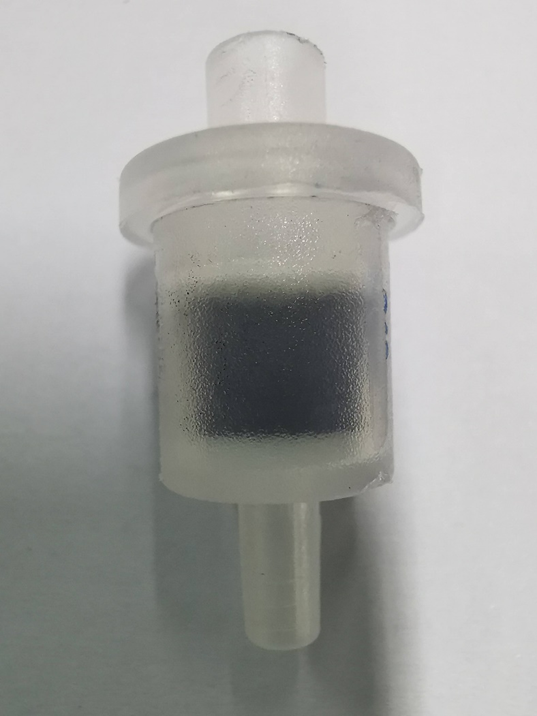


**Figure S9.** Photograph of a homemade short sorption column.

**Figure S10.** MDS of PFBA sorption by CF_700_.

**Figure S11.** MDS of PFOA sorption by CF_700_.


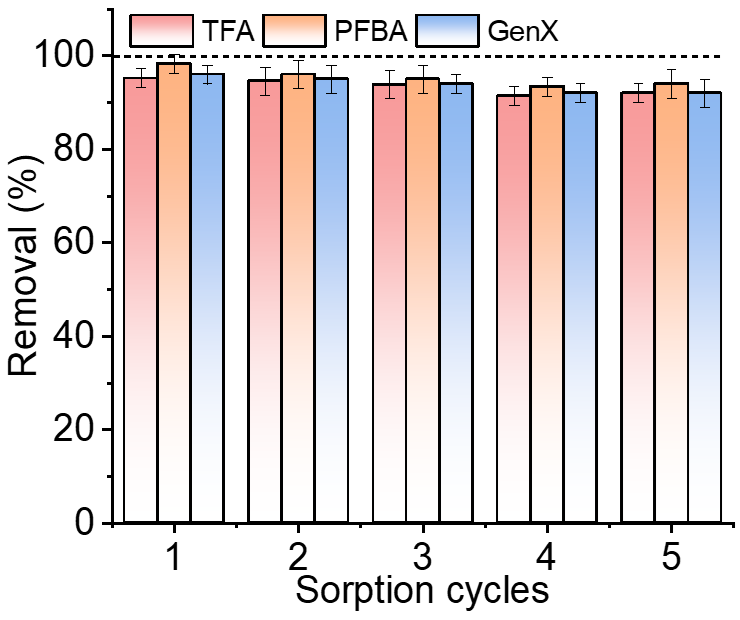


**Figure S12.** Regeneration ability of PPy_1_@P_0.5_-CF_700_ (single-solute system, [PFAS]_0_= 100 µg/L).

**Figure S13.** Chromatograms and MS/MS spectrum of [C_2_F_5_COO]^-^ (m/z=162.9824) (Degradation time: 60 min; mass tolerance: 5 ppm).


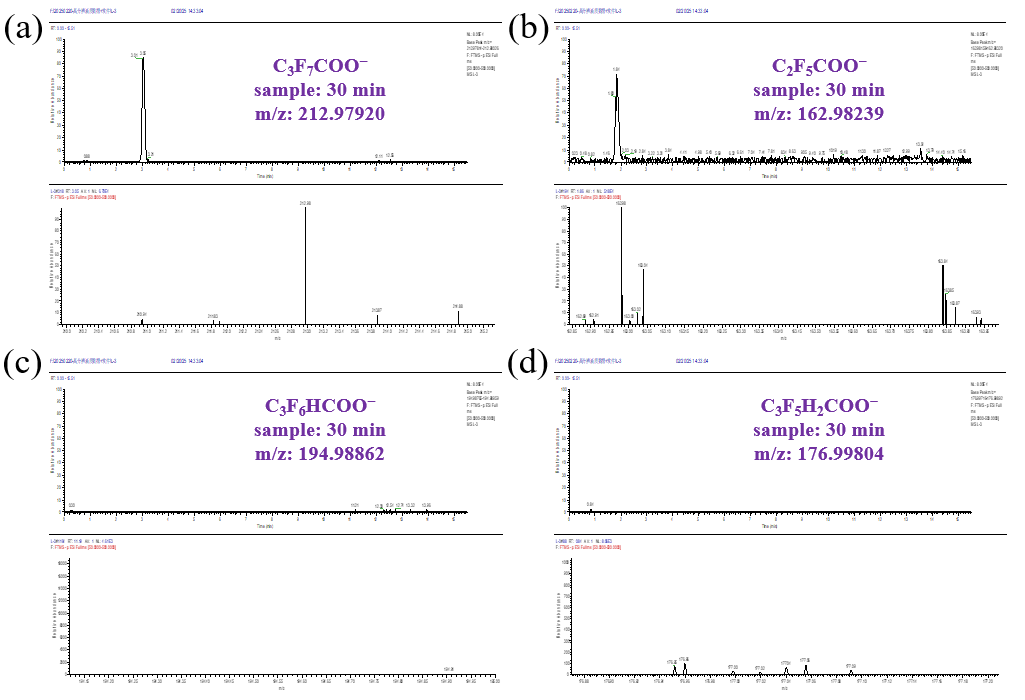


**Figure S14.** Searched for chain-shortening and H/F exchange fluorinated intermediates (Degradation time: 60 min; mass tolerance: 5 ppm).


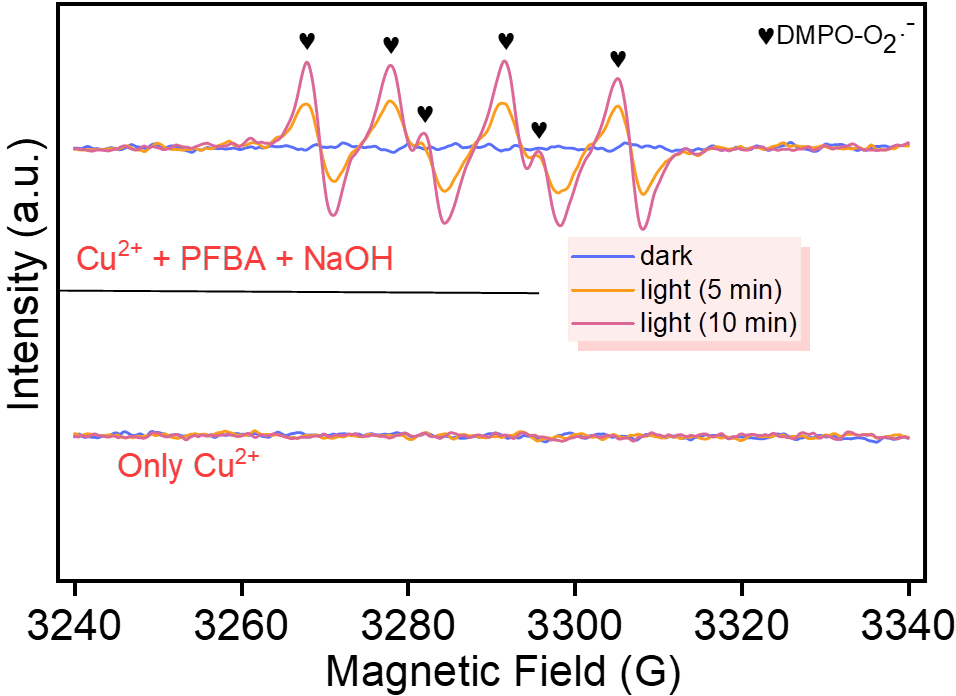


**Figure S15.** ESR spectra of DMPO-O_2_•^−^.

**Reference**

[1] H. Sun, F. S. Cannon, X. He, *Sci. Total Environ.* **2019**, 660, 577.

[2] J. Zhou, N. Saeidi, L. Y. Wick, Y. Xie, F.-D. Kopinke, A. Georgi, *J. Hazard. Mater.* **2022**, 436, 129051.

[3] Z. Du, S. Deng, Y. Chen, B. Wang, J. Huang, Y. Wang, G. Yu, *J. Hazard. Mater.* **2015**, 286, 136.

[4] P. Meng, X. Fang, A. Maimaiti, G. Yu, S. Deng, *Chemosphere* **2019**, 224, 187.

[5] M. Inyang, E. R. V. Dickenson, *Chemosphere* **2017**, 184, 168.

[6] N. Liu, C. Wu, G. Lyu, M. Li, *Sci. Total Environ.* **2021**, 798.

[7] D. Zhang, Q. He, M. Wang, W. Zhang, Y. Liang, *Environ. Technol.* **2019**, 42, 1798.

[8] S. S. Dalahmeh, N. Alziq, L. Ahrens, *Environ. Pollut.* **2019**, 247, 155.

[9] A. Maimaiti, S. Deng, P. Meng, W. Wang, B. Wang, J. Huang, Y. Wang, G. Yu, *Chem. Eng. J.* **2018**, 348, 494.

[10] Q. Yu, R. Zhang, S. Deng, J. Huang, G. Yu, *Water Res.* **2009**, 43, 1150.

[11] X. Tan, P. Dewapriya, P. Prasad, Y. Chang, X. Huang, Y. Wang, X. Gong, T. E. Hopkins, C. Fu, K. V. Thomas, H. Peng, A. K. Whittaker, C. Zhang, *Angew. Chem. Int. Ed.* **2022**, 61, e202213071.

[12] W. Ji, L. Xiao, Y. Ling, C. Ching, M. Matsumoto, R. P. Bisbey, D. E. Helbling, W. R. Dichtel, *J. Am. Chem. Soc.* **2018**, 140, 12677.

[13] Z. Yang, Y. Zhu, X. Tan, S. Gunjal, P. Dewapriya, Y. Wang, R. Xin, C. Fu, K. Liu, K. Macintosh, L. Sprague, L. Leung, T. Hopkins, K. Thomas, J. Guo, A. Whittaker, C. Zhang, *Nature Commun.* **2024**, 15, 8269.

[14] E. Loukopoulos, S. Marugán-Benito, D. Raptis, E. Tylianakis, G. Froudakis, A. Mavrandonakis, A. Platero-Prats, *Adv. Funct. Mater.* **2024**, 34, 2409932.

[15] J. Huang, Y. Shi, J. Xu, J. Zheng, F. Zhu, X. Liu, G. Ouyang, *Adv. Funct. Mater.* **2022**, 2203171.

[16] W. Wang, Z. Zhou, H. Shao, S. Zhou, G. Yu, S. Deng, *Chem. Eng. J.* **2021**, 412, 127509.

[17] D. Song, B. Qiao, Y. Yao, L. Zhao, X. Wang, H. Chen, L. Zhu, H. Sun, *J. Hazard. Mater.* **2023**, 460, 132411.
